# Organic–inorganic hybrid mixed-halide Zn^II^ and Cd^II^ tetra­halometallates with the 2-methyl­imidazo[1,5-*a*]pyridinium cation

**DOI:** 10.1107/S2056989022002420

**Published:** 2022-03-08

**Authors:** Olga Yu. Vassilyeva, Elena A. Buvaylo, Vladimir N. Kokozay, Brian W. Skelton

**Affiliations:** a Department of Chemistry, Taras Shevchenko National University of Kyiv, 64/13 Volodymyrska Street, Kyiv 01601, Ukraine; b School of Molecular Sciences, University of Western Australia (M310), Perth, WA 6009, Australia

**Keywords:** crystal structure, disorder, tetra­hedral Zn^II^ and Cd^II^ anions, halide substitution, π-bonded chains, NMR spectra

## Abstract

Three isomorphous 0-D hybrid salts, namely, 2-methyl­imidazo[1,5-*a*]pyridinium tri­chlorido­iodido­zincate(II), (C_8_H_9_N_2_)_2_[ZnCl_3.19_I_0.81_] or [*L*]_2_[ZnCl_3.19_I_0.81_], (I), 2-methyl­imidazo[1,5-*a*]pyridinium di­bromido­dichlorido­cadmate(II), (C_8_H_9_N_2_)_2_[CdBr_2.42_Cl_1.58_] or [*L*]_2_[CdBr_2.42_Cl_1.58_], (II), and 2-methyl­imidazo[1,5-*a*]pyridinium tri­chlorido­iodido­cadmate(II), (C_8_H_9_N_2_)_2_[CdCl_3.90_I_0.10_] or [*L*]_2_[CdCl_3.90_I_0.10_], (III), are assembled from discrete 2-methyl­imidazo[1,5-*a*]pyridinium cations, *L* ^+^, and mixed-halide tetra­halometallate anions. In the three structures, there are two crystallographically non-equivalent cations that were modelled as being rotationally disordered by 180°. In the lattices of the three compounds, a disordered state exists involving partial substitution of Cl by I for sites 2–4 in (I), Br by Cl for all four sites in (II) and Cl by I for site 2 in (III). In the solid state, the organic and inorganic sheets alternate parallel to the *bc* plane in a pseudo-layered arrangement. In the organic layer, pairs of centrosymmetic­ally related *trans*-oriented cations form π-bonded chains. The adjacent tetra­halometallate anions in the inorganic layer show no connectivity with the shortest *M*⋯*M* separations being greater than 7 Å. A variety of C—H⋯*X*—*M* (*X* = Cl, Br, I) contacts between the organic and inorganic counterparts provide additional structural stabilization. The title structures are isomorphous with the previously reported structures of the chloride analogues, [*L*]_2_[ZnCl_4_] and [*L*]_2_[CdCl_4_].

## Abstract 2

The three isomorphous hybrid salts are assembled from discrete 2-methyl­imidazo[1,5-*a*]pyridinium cations and Zn^II^ or Cd^II^ tetra­halometallate anions that show disorder involving partial substitution of Br by Cl and Cl by I in the [CdBr_2.42_Cl_1.58_]^2–^, [CdCl_3.90_I_0.10_]^2–^ and [ZnCl_3.19_I_0.81_]^2–^ anions.

## Chemical context

Hybrid organic–inorganic halide salts have proven to be promising materials for optoelectronic applications spanning light-emitting diodes (LED), lasers, photodetectors and solar cells (Manser *et al.*, 2016 ▸; Dou *et al.*, 2014 ▸; Stranks *et al.*, 2015 ▸). The versatile photophysical properties of these materials are combined with low-temperature solution processability and the tunability of their electronic and crystal structures *via* chemical composition modification. This research field has been mostly dominated by Pb- and Sn-based hybrid halide perovskites due to their prominent semiconducting properties and large optical absorption. However, water permeability in air and the low thermal stability of these perovskite systems limit their industrial manufacturing (Leijtens *et al.*, 2015 ▸). The instability issues have been largely related to the volatility of small organic cations. The introduction of larger organic cations that also lower the dimensionality of a 3-D *MX*_6_ (*X* = halide ion) octa­hedral halometallate network is expected to improve the air, moisture and thermal stability of the hybrid metal halides (Leblanc *et al.*, 2019 ▸).

The selective combination of organic and inorganic components to incorporate other metal polyhedra and connectivity directly impacts the properties exhibited by the organic–inorganic halide materials. Hybrid halometallates containing group 12 (II*B*) elements have been of increasing research inter­est in this respect (Yangui *et al.*, 2019 ▸). Based on the combined experimental and computational results, (CH_3_NH_3_)_2_Cd*X*_4_ (*X* = Cl, Br, I) and related compounds were found to be potential candidates for broadband white-light emitting phosphors and self-activated scintillators (Roccanova *et al.*, 2017 ▸). Engineering hybrid halometallate salts through mixing halogen elements is a recent new strategy that allows fine-tuning of the electronic structure and optoelectronic properties depending on the anionic speciation and ratio (Askar *et al.*, 2018 ▸; Rogers *et al.*, 2019 ▸).

Recently, we have developed a successful synthetic procedure towards organic–inorganic hybrid halometallates with imidazo[1,5-*a*]pyridinium-based counter-ions (Buvaylo *et al.*, 2015 ▸; Vassylyeva *et al.*, 2020 ▸). The latter represent an important class of fused nitro­gen-containing bicyclic systems owing to their biological activity and potential applications in materials chemistry. They show strong fluorescence intensity and high quantum yield (Yagishita *et al.*, 2018 ▸). The 2-meth­yl­imidazo[1,5-*a*] pyridinium cation, *L* ^+^, has been synthesized from the oxidative cyclo­condensation of equimolar amounts of formaldehyde, methyl­amine hydro­chloride and 2-pyrid­ine­carbaldehyde in an aqueous solution. The incorporation of *L* ^+^ in the metal chloride structure reduced the dimensionality of the PbCl_2_ 3-D perovskite framework to a 1-D stepwise chloro­plumbate(II) wire arrangement in [*L*]_*n*_[PbCl_3_]_*n*∞_ and produced [*L*]_2_[*M*Cl_4_] (*M* = Zn, Cd) hybrid salts with tetra­hedral anions (Vassilyeva *et al.*, 2020 ▸, 2021 ▸). The three compounds exhibited intense sky blue-light photoluminescence in the solid state. 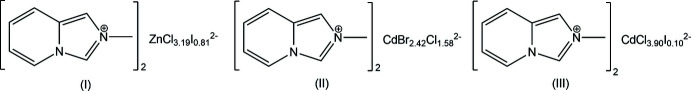


In this work, we have explored the possibility of preparing the Br and I analogues of [*L*]_2_[*M*Cl_4_] hybrids in an attempt to induce changes of the dimensionality in the resulting structures. In the synthesis, a combination of ZnO and NH_4_I was used instead of ZnCl_2_, while cadmium chloride was replaced with the corresponding bromide or iodide. This approach appeared to be only partially successful because of the competing Cl^−^ anions from the dissociation of the HCl adduct of methyl­amine. Herein, we report the preparations, crystal structures and spectroscopic characterization of three isomorphous 0-D hybrid salts [*L*]_2_[ZnCl_3.19_I_0.81_], (I), [*L*]_2_[CdBr_2.42_Cl_1.58_], (II), and [*L*]_2_[CdCl_3.90_I_0.10_], (III).

## Structural commentary

**Figure 1:**
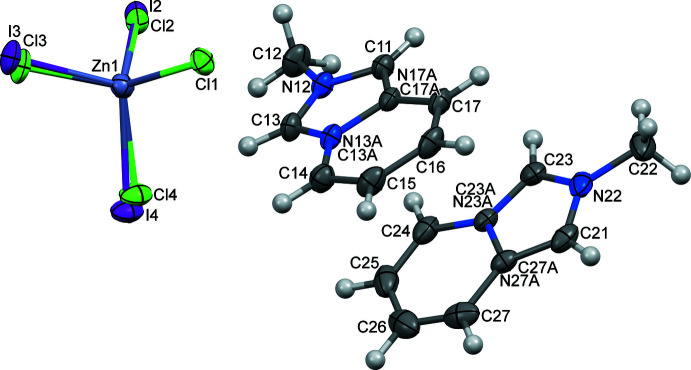
Mol­ecular structure of [*L*]_2_[ZnCl_3.19_I_0.81_], (I), with 50% probability displacement ellipsoids showing the general geometry and atom labelling of the three hybrid salts.

The organic—inorganic hybrids (I)–(III) crystallize in the triclinic space group *P* 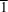 and are assembled from discrete 2-methyl­imidazo[1,5-*a*]pyridinium cations and mixed-halide tetra­halometallate anions. Fig. 1 ▸ shows the mol­ecular structure and labelling of (I) taken as a representative example. In the three structures, there are two crystallographically non-equivalent cations (*L*1^+^ and *L*2^+^) with similar structural configurations, which do not differ significantly from those of the isomorphous sister compounds [*L*]_2_[ZnCl_4_] (GOTHAB; Vassilyeva *et al.*, 2020 ▸) and [*L*]_2_[CdCl_4_] (GOTJAD; Vassilyeva *et al.*, 2021 ▸). The C—N/C bond distances in the imidazolium entities of the fused cores of the cations vary in the range 1.332 (3)–1.408 (4) Å; bond lengths in the pyridinium rings are as expected; the nitro­gen atoms are planar with the sums of the three angles being equal to 360°. The almost coplanar five- and six-membered rings in the cations show dihedral angles between them of about 2° [(I): 0.57 (13), 2.11 (12)°; (II): 0.73 (14), 1.55 (15)°; (III): 0.55 (16), 1.66 (17)°]. The tetra­hedral Zn*X*_4_^2–^ and Cd*X*_4_^2–^ (*X* = Cl, Br, I) anions in the hybrid salts are slightly distorted with the *M*—*X* distances falling in the ranges 2.2689 (10)–2.5969 (4), 2.380 (4)–2.6029 (11) and 2.4481 (8)–2.747 (4) Å for (I), (II) and (III), respectively (Tables 1 ▸–3 ▸ ▸). The *X*—*M*—*X* angles vary from 104.9 (5) to 117.3 (5)°. In the lattices of the three hybrid salts, a disordered state exists involving partial substitution of Cl by I for sites 2–4 in (I), Br by Cl for all four sites in (II) and Cl by I for site 2 in (III). Such a disorder occurs frequently in compounds containing two different halide ions resulting from the competition between them during the crystals formation (Yang *et al.*, 2010 ▸). The Zn—Cl and Cd—Cl bond lengths in (I)–(III) are similar to those of GOTHAB [2.2682 (4)–2.2920 (4) Å] and GOTJAD [2.4477 (5)–2.4719 (5) Å].

## Supra­molecular features

**Figure 2:**
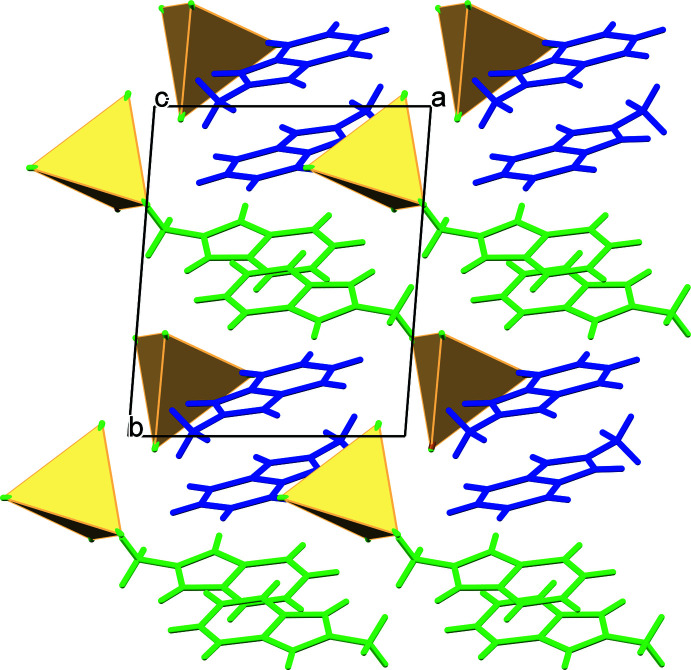
Fragment of the crystal packing of (II) viewed along the *c* axis with the non-equivalent *L*1^+^ and *L*2^+^ cations shown in blue and green, respectively, and [CdBr_2.42_Cl_1.58_]^2–^ anions presented in polyhedral form.

**Figure 3:**
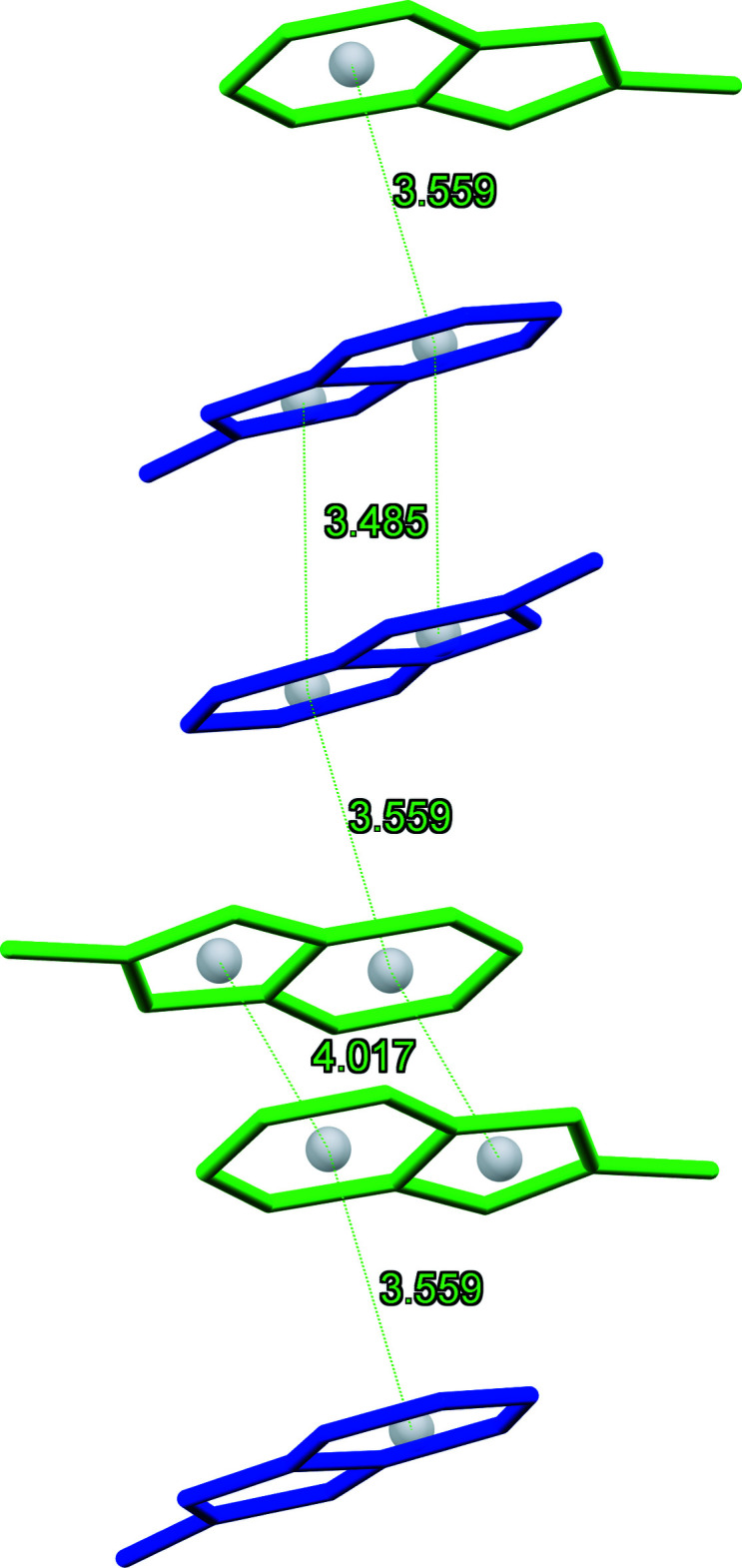
Fragment of the π-bonded chain built of pairs of the non-equivalent *L*1^+^ and *L*2^+^ cations of [*L*]_2_[CdCl_3.90_I_0.10_] (III).

In the crystals of (I)–(III), the organic and inorganic sheets alternate parallel to the *bc* plane in a pseudo-layered arrangement. Fig. 2 ▸ illustrates the crystal packing common for the three compounds. The consecutive inorganic planes are separated by a distance corresponding to the *a*-axis length [9.4588 (6), 9.5172 (5) and 9.4304 (3) Å for (I)–(III), respectively]. In the organic layer, pairs of centrosymmetically related *trans*-oriented *L*1^+^ and *L*2^+^ cations form π-bonded chains with the centroid–centroid distances between the pairs being 3.543 (2) Å in (I), 3.569 (2) Å in (II) and 3.559 (2) Å in (III) (Fig. 3 ▸). The pairs of equivalent cations in the chains demonstrate stronger and weaker 10π*e*–10π*e* stacking with the centroid–centroid distances for (I), (II) and (III) of 3.448 (2), 4.099 (2) Å; 3.496 (2), 4.105 (2) Å and 3.485 (2), 4.017 (2) Å, respectively. The adjacent tetra­halometallate anions in the inorganic layer show no connectivity with the shortest *M*⋯*M* separations being about 7.287 in (I), 7.158 in (II) and 7.046 Å in (III). In the hybrid salts, classical hydrogen bonds are absent. A variety of C—H⋯*X*—*M* contacts (see supporting information) between the organic and inorganic counterparts with the H⋯*X* distances below the van der Waals contact limits of 2.85 (Cl), 2.93 (Br) and 3.08 Å (iodine) (Mantina *et al.*, 2009 ▸) provide an additional structure-stabilizing effect.

## Database survey

More than 300 crystal structures of mol­ecules featuring the imidazo[1,5-*a*]pyridine core are found in the CSD (Version 5.42, update of February 2021; Groom *et al.*, 2016 ▸). Those comprise neutral organic compounds, organic salts and metal complexes with the imidazo[1,5-*a*]pyridine core having various substituents in the rings. Apart from [*L*]_2_[CdCl_4_] (GOTJAD; Vassilyeva *et al.*, 2021 ▸), [*L*]_2_[ZnCl_4_] (GOTHAB; Vassilyeva *et al.*, 2020 ▸) and [*L*]_*n*_[PbCl_3_]_*n*∞_ (TURJUO; Vassilyeva *et al.*, 2020 ▸) published by our research group, there are no structures containing the *L* ^+^ cation in the Database. The reported compounds with cations similar to *L* ^+^ of the title hybrid salts are, for example, 2-(2,4,6-tri­methyl­phen­yl)-2*H*-imidazo[1,5-*a*]pyridin-4-ium bromide (PARBOA; Burstein *et al.*, 2005 ▸) and 2-(4-chloro­phen­yl)imidazo[1,5-*a*]pyridinium perchlorate (ETOXEQ; Chattopadhyay *et al.*, 2004 ▸) having tri­methyl­phenyl and chloro­phenyl substituents, respectively, instead of the methyl group in *L* ^+^. Such organic cations are precursors for *N*-heterocyclic carbenes, which are able to bind metal ions as in *e.g*. bis­(2-*t*-butyl­imidazo[1,5-*a*]pyridin-3-yl­idene)(η^4^-1,5- cyclo­octa­diene)rhodium(I) hexa­fluoro­phosphate (FOJYAF; Alcarazo *et al.*, 2005 ▸) or bis­[2-(2-pyrid­yl)imidazo[1,5-*a*]pyridin-3(2*H*)-yl­idene]mercury bis­(hexa­fluoro­phosphate) (IVOWEW; Samanta *et al.*, 2011 ▸). The neutral derivatives of the *L* ^+^ cation lacking the methyl group but possessing other substituents with donor atoms (N, O, S) often act as ligands that coordinate various metal ions: chloro-bis­[3-(pyridin-2-yl)imidazo[1,5-*a*]pyridine]­copper(II) chloride ethanol solvate (ELILOD; Carson *et al.*, 2021 ▸) or bis­[2-(1-phenyl­imidazo[1,5-a]pyridin-3-yl)phenolato]cobalt(II) 1,2-di­chloro­ethane solvate (KESQUX; Ardizzoia *et al.*, 2018 ▸).

## FTIR and ^1^NMR spectroscopy

**Figure 4:**
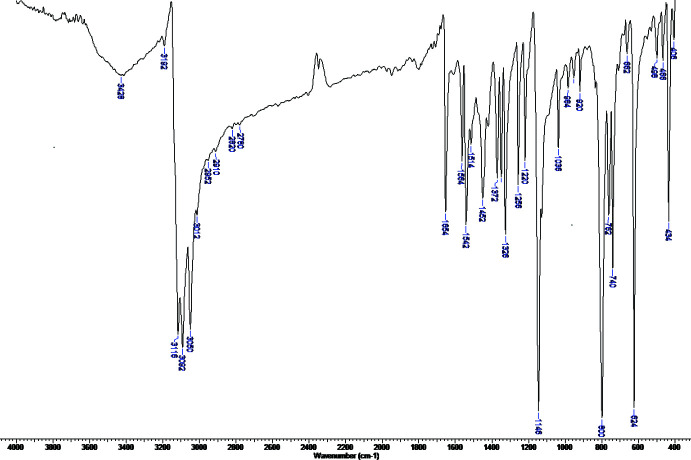
IR spectrum of [*L*]_2_[CdBr_2.42_Cl_1.58_], (II).

The very similar IR spectra of hybrid salts (I)–(III) show a distinctive pattern we consider characteristic of the *L* ^+^ cation (Vassilyeva *et al.*, 2020 ▸) (Fig. 4 ▸). The spectra are distinguished by the very sharp intense peaks in the aromatic ν(C—H) stretching region (3130–3012 cm^−1^) and the lack of absorbance from 1656 to 1568 cm^−1^. They include weak bands below 3000 cm^−1^ due to alkyl C—H stretching, sharp bands of medium intensity at 1654/1654/1656, 1542/1542/1546, 1450/1452/1456 and 1328/1326/1332 cm^−1^ associated with heterocyclic rings stretching, a very strong band at 1150/1146/1152 cm^−1^ ascribed to ν(N–C_CH3_) vibration and a noticeable set of three very intense absorptions in the out-of-plane C—H bending region 800–600 cm^−1^ (peaks at 789/800/780, 738/740/734 and 616/624/618 cm^−1^) for (I)/(II)/(III), respectively.

**Figure 5:**
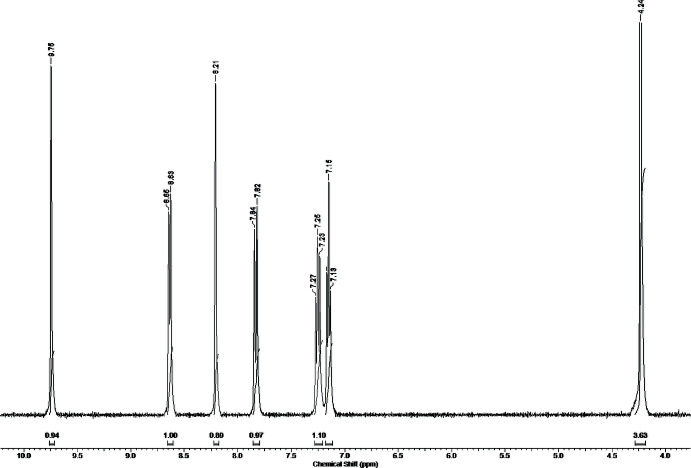
The room-temperature ^1^H NMR spectrum of (III) in DMSO-*d*_6_ in the 10–4 ppm range.

The room-temperature ^1^H NMR spectra of the hybrids in DMSO-*d*_6_ are similar, demonstrating the expected sets of signals and correct aromatic/alkyl proton ratios of the *L* ^+^ cation (Fig. 5 ▸). Two CH protons in the imidazolium rings appear as singlets at δ 9.88/9.75/9.81 [H_C13_] and 8.25/8.21/8.22 ppm [H_C11_] for (I)/(II)/(III), respectively. The pyridine protons give two doublet and two triplet resonances between 8.67/8.64/8.68 and 7.11/7.15/7.14 ppm. Protons of the CH_3_ group are observed as singlets at 4.26/4.24/4.25 ppm. The close resemblance of the measured ^1^H NMR spectra with those of other *L* ^+^-containing halometallates (Vassilyeva *et al.*, 2020 ▸, 2021 ▸) implies that the *L* ^+^ cation is conformationally stable in solutions of both hybrid salts, which are thus dissociated in DMSO.

## Synthesis and crystallization

**Synthesis of [** ***L*** **]_2_[ZnCl_3.19_I_0.81_] (I)**

Solid CH_3_NH_2_·HCl (0.27 g, 4 mmol) was added to the warm formaldehyde solution prepared by dissolving paraform (0.13 g, 4.5 mmol) in boiling deionized water (10 ml) in a 50 ml conical flask. The solution was stirred vigorously for 1 h at room temperature, filtered, and 2-pyridine­carbaldehyde (0.19 ml, 2 mmol) was added to the flask, which was then left open overnight. On the following day, ZnO (0.08 g, 1 mmol) and NH_4_I (0.29 g, 2 mmol) were introduced into the flask and the mixture was magnetically stirred at 323 K for 1.5 h. After that, the turbid orange solution was filtered and allowed to evaporate. Very light brownish prisms of (I) suitable for X-ray crystallography formed within two weeks in the brown solution. The crystals were filtered off, washed with diethyl ether and dried in air. Yield: 83% (based on Zn). FT–IR (ν, cm^−1^): 3436*br*, 3114*s*, 3094*vs*, 3068, 3038*vs*, 3006, 2972, 2934, 1654, 1562, 1542, 1450, 1376, 1346, 1322, 1262, 1216, 1150*vs*, 1128, 1036, 986, 918, 789*vs*, 762, 738, 616*vs*, 500, 466, 424. ^1^H NMR (400MHz, DMSO-*d*_6_): δ (ppm) 9.88 (*s*, 1H, H_C13_), 8.67 (*d*, 1H, *J* = 6.9 Hz, H_C14_), 8.25 (*s*, 1H, H_C11_), 7.80 (*d*, 1H, *J* = 9.2 Hz, H_C17_), 7.21 (*t*, 1H, *J* = 8.1 Hz, H_C15_), 7.11 (*t*, 1H, *J* = 6.7 Hz, H_C16_), 4.26 (*s*, 3H, CH_3_). Analysis calculated for C_16_H_18_N_4_ZnCl_3_I (564.99): C 34.01; H 3.21; N 9.92%. Found: C 35.40; H 2.83; N 9.74%.

**Synthesis of [** ***L*** **]_2_[CdBr_2.42_Cl_1.58_] (II)**

The compound was prepared by a similar procedure except that CdBr_2_·4H_2_O (0.34 g, 1 mmol) dissolved in water was used instead of ZnO and NH_4_I. Yield: 72% (based on cadmium). FT–IR (ν, cm^−1^): 3428*br*, 3116*s*, 3092*s*, 3050*s*, 3012, 2952, 2910, 1654, 1564, 1542, 1452, 1372, 1350, 1326, 1256, 1220, 1146*vs*, 1036, 984, 920, 800*vs*, 762, 740, 624*vs*, 498, 466, 434, 406. ^1^H NMR (400 MHz, DMSO-*d*_6_): δ (ppm) 9.81 (*s*, 1H, H_C13_), 8.68 (*d*, 1H, *J* = 6.8 Hz, H_C14_), 8.22 (*s*, 1H, H_C11_), 7.83 (*d*, 1H, *J* = 9.3 Hz, H_C17_), 7.24 (*t*, 1H, *J* = 8.1 Hz, H_C15_), 7.14 (*t*, 1H, *J* = 6.8 Hz, H_C16_), 4.25 (*s*, 3H, CH_3_). Analysis calculated for C_16_H_18_N_4_CdBr_3_Cl (653.92): C 29.39; H 2.77; N 8.57%. Found: C 28.91; H 2.84; N 8.68%.

**Synthesis of [** ***L*** **]_2_[CdCl_3.90_I_0.10_] (III)**

The compound was synthesized in a similar way by employing CdI_2_ (0.36 g, 1 mmol) dissolved in water in place of ZnO and NH_4_I. Yield: 89% (based on cadmium). FT–IR (ν, cm^−1^): 3420*br*, 3130*s*, 3098*s*, 3072, 3054, 2990, 2944, 2914, 1656, 1568, 1546, 1456, 1376, 1356, 1332, 1256, 1218, 1152s, 1040, 982, 920, 780*vs*, 734, 618s, 504, 464, 432, 418. ^1^H NMR (400 MHz, DMSO-*d*_6_): δ (ppm) 9.75 (*s*, 1H, H_C13_), 8.64 (*d*, 1H, *J* = 7.3 Hz, H_C14_), 8.21 (*s*, 1H, H_C11_), 7.83 (*d*, 1H, *J* = 9.3 Hz, H_C17_), 7.25 (*t*, 1H, *J* = 7.8 Hz, H_C15_), 7.15 (*t*, 1H, *J* = 7.1 Hz, H_C16_), 4.24 (*s*, 3H, CH_3_). Analysis calculated for C_16_H_18_N_4_ZnClI_3_ (794.92): C, 25.69; H 2.43; N 7.49%. Found: C 22.74; H 1.79; N 6.42%. The iodine content in the bulk sample appeared significantly larger than in the single crystal of (III) used for data collection.

## Refinement

Crystal data, data collection and structure refinement details are summarized in Table 4 ▸. In all three structures, the cations were modelled as being rotationally disordered by 180°. The site occupancies refined to 0.855 (17) and its complement for both cations in (I), 0.73 (2) and its complement for cation 1 and 0.75 (2) and its complement for cation 2 in (II), and 0.72 (3) and its complement for cation 1 and 0.81 (3) and its complement for cation 2 in (III). In compound (I), the halide atom sites 2, 3 and 4 were modelled as being part Cl and part I, with Cl site occupancies refined to 0.3034 (15), 0.9489 (12) and 0.9343 (12), respectively, with the I site occupancies being the complements. The halide atom sites in compound (II) were modelled as being part Br and part Cl with the Br occupancy for sites 1–4 refined to 0.417 (2), 0.857 (2), 0.558 (2) and 0.590 (2) with the Cl occupancies being the complements. Cd—*X* bond lengths of the disordered atoms were restrained to ideal values. The halide atom site 2 in (III) was modelled as being part Cl and part I, with Cl site occupancies refined to 0.9008 (15) with the I site occupancies being its complement. Cd–X bond lengths of the disordered atoms were restrained to ideal values. The coordinates of the halogens were refined to be independent for all three structures. All hydrogen atoms were included in calculated positions and refined using a riding model with isotropic displacement parameters based on those of the parent atom (C—H = 0.95 Å, *U*_iso_(H) = 1.2*U*_eq_(C) for CH, C—H = 0.98 Å, *U*_iso_(H) = 1.5*U*_eq_(C) for CH_3_). Anisotropic displacement parameters were employed for the non-hydrogen atoms.

## Supplementary Material

Crystal structure: contains datablock(s) I, II, III, global. DOI: 10.1107/S2056989022002420/zn2015sup1.cif

Structure factors: contains datablock(s) I. DOI: 10.1107/S2056989022002420/zn2015Isup2.hkl

Structure factors: contains datablock(s) II. DOI: 10.1107/S2056989022002420/zn2015IIsup3.hkl

Structure factors: contains datablock(s) III. DOI: 10.1107/S2056989022002420/zn2015IIIsup4.hkl

CCDC references: 1960270, 1960327, 1960296

Additional supporting information:  crystallographic information; 3D view; checkCIF report

## Figures and Tables

**Table 1:** Selected geometric parameters (Å, °) for (I)

Zn1—Cl3	2.2689 (10)	Zn1—I3	2.542 (4)
Zn1—Cl4	2.2780 (11)	Zn1—I4	2.568 (3)
Zn1—Cl1	2.2884 (6)	Zn1—I2	2.5969 (4)
Zn1—Cl2	2.346 (3)		
			
Cl3—Zn1—Cl4	112.60 (5)	Cl3—Zn1—I4	107.23 (14)
Cl3—Zn1—Cl1	108.40 (4)	Cl1—Zn1—I4	109.51 (13)
Cl4—Zn1—Cl1	107.71 (4)	Cl2—Zn1—I4	110.37 (19)
Cl3—Zn1—Cl2	110.84 (13)	I3—Zn1—I4	110.4 (2)
Cl4—Zn1—Cl2	106.83 (14)	Cl3—Zn1—I2	109.83 (4)
Cl1—Zn1—Cl2	110.41 (12)	Cl4—Zn1—I2	106.78 (4)
Cl4—Zn1—I3	115.8 (2)	Cl1—Zn1—I2	111.54 (2)
Cl1—Zn1—I3	106.36 (19)	I3—Zn1—I2	108.8 (2)
Cl2—Zn1—I3	109.7 (2)	I4—Zn1—I2	110.22 (13)

**Table 2:** Selected geometric parameters (Å, °) for (II)

Cd1—Cl3	2.380 (4)	Cd1—Br3	2.5353 (12)
Cd1—Cl2	2.460 (5)	Cd1—Br1	2.5834 (17)
Cd1—Cl1	2.467 (3)	Cd1—Br2	2.5950 (5)
Cd1—Cl4	2.497 (4)	Cd1—Br4	2.6029 (11)
			
Cl3—Cd1—Cl2	107.3 (10)	Br3—Cd1—Br1	106.2 (3)
Cl3—Cd1—Cl1	106.1 (7)	Cl3—Cd1—Br2	108.3 (5)
Cl2—Cd1—Cl1	114.9 (9)	Cl1—Cd1—Br2	112.8 (5)
Cl3—Cd1—Cl4	112.7 (5)	Cl4—Cd1—Br2	110.6 (2)
Cl2—Cd1—Cl4	109.6 (9)	Br3—Cd1—Br2	109.34 (14)
Cl1—Cd1—Cl4	106.3 (6)	Br1—Cd1—Br2	111.3 (3)
Cl2—Cd1—Br3	108.4 (9)	Cl3—Cd1—Br4	117.3 (5)
Cl1—Cd1—Br3	105.3 (5)	Cl2—Cd1—Br4	106.6 (9)
Cl4—Cd1—Br3	112.4 (2)	Cl1—Cd1—Br4	104.9 (5)
Cl3—Cd1—Br1	107.0 (5)	Br3—Cd1—Br4	117.00 (15)
Cl2—Cd1—Br1	113.4 (8)	Br1—Cd1—Br4	105.4 (3)
Cl4—Cd1—Br1	106.9 (4)	Br2—Cd1—Br4	107.57 (8)

**Table 3:** Selected geometric parameters (Å, °) for (III)

Cd1—Cl3	2.4481 (8)	Cd1—Cl1	2.4710 (7)
Cd1—Cl2	2.4654 (16)	Cd1—I2	2.747 (4)
Cd1—Cl4	2.4655 (7)		
			
Cl3—Cd1—Cl2	109.94 (9)	Cl4—Cd1—Cl1	105.20 (3)
Cl3—Cd1—Cl4	116.91 (3)	Cl3—Cd1—I2	109.2 (2)
Cl2—Cd1—Cl4	106.67 (9)	Cl4—Cd1—I2	106.7 (2)
Cl3—Cd1—Cl1	105.93 (3)	Cl1—Cd1—I2	113.0 (2)
Cl2—Cd1—Cl1	112.21 (9)		

**Table 4:** Experimental details

	(I)	(II)	(III)
Crystal data			
Chemical formula	(C_8_H_9_N_2_)_2_[ZnCl_3.19_I_0.81_]	(C_8_H_9_N_2_)_2_[CdBr_2.42_Cl_1.58_]	(C_8_H_9_N_2_)_2_[CdCl_3.90_I_0.10_]
*M*_r_	547.59	628.14	529.69
Crystal system, space group	Triclinic, *P* 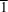	Triclinic, *P* 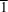	Triclinic, *P* 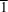
Temperature (K)	100	100	100
*a*, *b*, *c* (Å)	9.4588 (6), 10.8892 (8), 10.8343 (9)	9.5172 (5), 10.8293 (6), 10.9697 (6)	9.4304 (3), 10.7968 (3), 10.7565 (3)
α, β, γ (°)	100.305 (7), 110.910 (7), 90.955 (6)	99.620 (5), 110.413 (5), 90.827 (5)	99.209 (3), 110.746 (3), 90.837 (2)
*V* (Å^3^)	1021.67 (14)	1041.45 (10)	1007.97 (5)
*Z*	2	2	2
Radiation type	Mo *K*α	Mo *K*α	Cu *K*α
μ (mm^−1^)	2.85	5.90	14.69
Crystal size (mm)	0.68 × 0.48 × 0.20	0.36 × 0.28 × 0.11	0.25 × 0.08 × 0.04

Data collection			
Diffractometer	Oxford Diffraction Xcalibur diffractometer	Oxford Diffraction Gemini diffractometer	Oxford Diffraction Gemini diffractometer
Absorption correction	Analytical *CrysAlis PRO* (Rigaku OD, 2016 ▸)	Analytical *CrysAlis PRO* (Rigaku OD, 2016 ▸)	Analytical *CrysAlis PRO* (Rigaku OD, 2016 ▸)
*T*_min_, *T*_max_	0.284, 0.592	0.206, 0.53	0.052, 0.522
No. of measured, independent and observed [*I* > 2σ(*I*)] reflections	21436, 10105, 8082	15893, 6879, 5371	18506, 3581, 3309
*R*_int_	0.028	0.036	0.041
(sin θ/λ)_max_ (Å^−1^)	0.852	0.760	0.598

Refinement			
*R*[*F* ^2^ > 2σ(*F* ^2^)], *wR*(*F* ^2^), *S*	0.044, 0.104, 1.03	0.036, 0.068, 1.04	0.027, 0.068, 1.06
No. of reflections	10105	6879	3581
No. of parameters	241	246	234
No. of restraints	6	8	2
H-atom treatment	H-atom parameters constrained	H-atom parameters constrained	H-atom parameters constrained
Δρ_max_, Δρ_min_ (e Å^−3^)	1.67, −1.13	0.89, −0.77	0.79, −0.46

Computer programs: *CrysAlis PRO* (Rigaku OD, 2016 ▸), *SHELXT* (Sheldrick, 2015*a* ▸), *SHELXL2017* (Sheldrick, 2015*b* ▸), *Mercury* (Macrae *et al.*, 2020 ▸) and *WinGX* (Farrugia, 2012 ▸).

## supplementary crystallographic information

### Bis(2-methylimidazo[1,5-a]pyridinium) trichloridoiodidozincate(II), (I) Crystal data

**Table:**

(C_8_H_9_N_2_)_2_[ZnCl_3.19_I_0.81_]	*Z* = 2
*M**_r_* = 547.59	*F*(000) = 538
Triclinic, *P*1	*D*_x_ = 1.780 Mg m^−^^3^
*a* = 9.4588 (6) Å	Mo *K*α radiation, λ = 0.71073 Å
*b* = 10.8892 (8) Å	Cell parameters from 6804 reflections
*c* = 10.8343 (9) Å	θ = 2.1–36.7°
α = 100.305 (7)°	µ = 2.85 mm^−^^1^
β = 110.910 (7)°	*T* = 100 K
γ = 90.955 (6)°	Prism, colourless
*V* = 1021.67 (14) Å^3^	0.68 × 0.48 × 0.20 mm

### Bis(2-methylimidazo[1,5-a]pyridinium) trichloridoiodidozincate(II), (I) Data collection

**Table:**

Oxford Diffraction Xcalibur diffractometer	10105 independent reflections
Graphite monochromator	8082 reflections with *I* > 2σ(*I*)
Detector resolution: 16.0009 pixels mm^-1^	*R*_int_ = 0.028
ω scans	θ_max_ = 37.3°, θ_min_ = 1.9°
Absorption correction: analytical CrysAlis Pro (Rigaku OD, 2016)	*h* = −15→16
*T*_min_ = 0.284, *T*_max_ = 0.592	*k* = −18→18
21436 measured reflections	*l* = −16→17

### Bis(2-methylimidazo[1,5-a]pyridinium) trichloridoiodidozincate(II), (I) Refinement

**Table:**

Refinement on *F*^2^	6 restraints
Least-squares matrix: full	Hydrogen site location: inferred from neighbouring sites
*R*[*F*^2^ > 2σ(*F*^2^)] = 0.044	H-atom parameters constrained
*wR*(*F*^2^) = 0.104	*w* = 1/[σ^2^(*F*_o_^2^) + (0.0358*P*)^2^ + 1.1052*P*] where *P* = (*F*_o_^2^ + 2*F*_c_^2^)/3
*S* = 1.03	(Δ/σ)_max_ = 0.001
10105 reflections	Δρ_max_ = 1.67 e Å^−^^3^
241 parameters	Δρ_min_ = −1.13 e Å^−^^3^

### Bis(2-methylimidazo[1,5-a]pyridinium) trichloridoiodidozincate(II), (I) Special details

**Table:**

Geometry. All esds (except the esd in the dihedral angle between two l.s. planes) are estimated using the full covariance matrix. The cell esds are taken into account individually in the estimation of esds in distances, angles and torsion angles; correlations between esds in cell parameters are only used when they are defined by crystal symmetry. An approximate (isotropic) treatment of cell esds is used for estimating esds involving l.s. planes.
Refinement. The halogen sites 2,3,4 were modelled as being part Cl and part I, with Cl site occupancies refined to 0.3034 (15), 0.9489 (12) and 0.9343 (12) respectively with the I site occupancies being the complements. The cations were modelled as being rotationally disordered by 180 degrees. The site occupancies refined to 0.855 (17) and its complement for both cations after independent refinement showed insignificant differences in the values for the two cations.

### Bis(2-methylimidazo[1,5-a]pyridinium) trichloridoiodidozincate(II), (I) Fractional atomic coordinates and isotropic or equivalent isotropic displacement parameters (Å^2^)

**Table:**

	*x*	*y*	*z*	*U*_iso_*/*U*_eq_	Occ. (<1)
C11	0.6600 (2)	0.6614 (2)	0.1111 (2)	0.0248 (4)	
H11	0.646426	0.711007	0.044607	0.030*	
N12	0.7941 (2)	0.62397 (18)	0.1886 (2)	0.0252 (4)	
C12	0.9412 (3)	0.6546 (3)	0.1804 (3)	0.0357 (5)	
H12A	0.946281	0.605948	0.096999	0.053*	
H12B	1.022688	0.634282	0.258022	0.053*	
H12C	0.953186	0.744278	0.180683	0.053*	
C13	0.7715 (2)	0.5545 (2)	0.2710 (2)	0.0263 (4)	
H13	0.847038	0.517102	0.333981	0.032*	
N13A	0.6223 (2)	0.54728 (18)	0.2485 (2)	0.0233 (4)	0.855 (17)
C13A	0.6223 (2)	0.54728 (18)	0.2485 (2)	0.0233 (4)	0.145 (17)
C14	0.5420 (3)	0.4899 (2)	0.3113 (3)	0.0303 (5)	
H14	0.592227	0.444246	0.379171	0.036*	
C15	0.3917 (3)	0.5003 (2)	0.2739 (3)	0.0334 (5)	
H15	0.335840	0.463471	0.317513	0.040*	
C16	0.3147 (3)	0.5662 (2)	0.1692 (3)	0.0324 (5)	
H16	0.208174	0.571172	0.143478	0.039*	
C17	0.3906 (2)	0.6215 (2)	0.1063 (3)	0.0273 (4)	
H17	0.338477	0.664132	0.036158	0.033*	
C17A	0.5491 (2)	0.61438 (19)	0.1470 (2)	0.0222 (4)	0.855 (17)
N17A	0.5491 (2)	0.61438 (19)	0.1470 (2)	0.0222 (4)	0.145 (17)
C21	0.2953 (3)	0.9033 (2)	0.3100 (3)	0.0312 (5)	
H21	0.197680	0.902835	0.316380	0.037*	
N22	0.3346 (2)	0.9439 (2)	0.2132 (2)	0.0312 (4)	
C22	0.2337 (4)	0.9986 (3)	0.1032 (3)	0.0476 (8)	
H22A	0.201246	1.076857	0.140803	0.071*	
H22B	0.144421	0.939600	0.049935	0.071*	
H22C	0.288028	1.015842	0.045426	0.071*	
C23	0.4809 (3)	0.9309 (2)	0.2357 (2)	0.0286 (4)	
H23	0.535049	0.952375	0.182601	0.034*	
N23A	0.5377 (2)	0.88212 (18)	0.34650 (19)	0.0228 (4)	0.855 (17)
C23A	0.5377 (2)	0.88212 (18)	0.34650 (19)	0.0228 (4)	0.145 (17)
C24	0.6857 (3)	0.8517 (2)	0.4121 (3)	0.0324 (5)	
H24	0.763660	0.864674	0.378530	0.039*	
C25	0.7145 (4)	0.8036 (3)	0.5241 (3)	0.0417 (7)	
H25	0.813962	0.781557	0.569003	0.050*	
C26	0.6008 (4)	0.7853 (3)	0.5760 (3)	0.0435 (7)	
H26	0.625542	0.752176	0.655778	0.052*	
C27	0.4577 (4)	0.8137 (2)	0.5151 (3)	0.0377 (6)	
H27	0.381785	0.800864	0.550997	0.045*	
C27A	0.4226 (3)	0.8635 (2)	0.3958 (2)	0.0254 (4)	0.855 (17)
N27A	0.4226 (3)	0.8635 (2)	0.3958 (2)	0.0254 (4)	0.145 (17)
Zn1	0.83992 (3)	0.19500 (3)	0.25185 (3)	0.02399 (7)	
Cl1	0.58526 (6)	0.19206 (6)	0.13225 (6)	0.02775 (11)	
Cl2	0.9808 (5)	0.2984 (5)	0.1553 (5)	0.02266 (7)	0.3034 (15)
I2	1.00082 (4)	0.30678 (4)	0.14728 (5)	0.02266 (7)	0.6966 (15)
Cl3	0.90002 (15)	−0.00570 (10)	0.25354 (14)	0.03234 (17)	0.9489 (12)
I3	0.8998 (9)	−0.0326 (4)	0.2395 (9)	0.03234 (17)	0.0511 (12)
Cl4	0.88891 (13)	0.30928 (11)	0.46268 (11)	0.03401 (18)	0.9343 (12)
I4	0.8998 (6)	0.3042 (5)	0.4976 (3)	0.03401 (18)	0.0657 (12)

### Bis(2-methylimidazo[1,5-a]pyridinium) trichloridoiodidozincate(II), (I) Atomic displacement parameters (Å^2^)

**Table:**

	*U*^11^	*U*^22^	*U*^33^	*U*^12^	*U*^13^	*U*^23^
C11	0.0214 (9)	0.0258 (10)	0.0284 (10)	0.0043 (7)	0.0106 (8)	0.0048 (8)
N12	0.0192 (8)	0.0252 (8)	0.0332 (10)	0.0036 (6)	0.0124 (7)	0.0046 (7)
C12	0.0237 (10)	0.0383 (13)	0.0512 (16)	0.0028 (9)	0.0209 (11)	0.0092 (11)
C13	0.0195 (9)	0.0253 (10)	0.0338 (11)	0.0055 (7)	0.0091 (8)	0.0063 (8)
N13A	0.0191 (8)	0.0209 (8)	0.0302 (9)	0.0031 (6)	0.0102 (7)	0.0030 (7)
C13A	0.0191 (8)	0.0209 (8)	0.0302 (9)	0.0031 (6)	0.0102 (7)	0.0030 (7)
C14	0.0308 (11)	0.0270 (11)	0.0370 (12)	0.0038 (8)	0.0162 (10)	0.0077 (9)
C15	0.0304 (12)	0.0281 (11)	0.0472 (15)	0.0001 (9)	0.0224 (11)	0.0042 (10)
C16	0.0203 (9)	0.0283 (11)	0.0483 (14)	0.0022 (8)	0.0161 (10)	−0.0006 (10)
C17	0.0186 (9)	0.0262 (10)	0.0330 (11)	0.0045 (7)	0.0076 (8)	−0.0008 (8)
C17A	0.0193 (8)	0.0194 (8)	0.0263 (9)	0.0037 (6)	0.0085 (7)	−0.0003 (7)
N17A	0.0193 (8)	0.0194 (8)	0.0263 (9)	0.0037 (6)	0.0085 (7)	−0.0003 (7)
C21	0.0229 (10)	0.0273 (11)	0.0381 (13)	−0.0006 (8)	0.0120 (9)	−0.0079 (9)
N22	0.0312 (10)	0.0267 (9)	0.0270 (9)	0.0090 (8)	0.0039 (8)	−0.0032 (7)
C22	0.0502 (17)	0.0425 (16)	0.0310 (13)	0.0197 (13)	−0.0042 (12)	−0.0023 (11)
C23	0.0346 (12)	0.0255 (10)	0.0258 (10)	0.0061 (8)	0.0135 (9)	0.0002 (8)
N23A	0.0224 (8)	0.0213 (8)	0.0246 (8)	0.0021 (6)	0.0109 (7)	−0.0006 (6)
C23A	0.0224 (8)	0.0213 (8)	0.0246 (8)	0.0021 (6)	0.0109 (7)	−0.0006 (6)
C24	0.0242 (10)	0.0300 (11)	0.0367 (12)	0.0039 (8)	0.0096 (9)	−0.0058 (9)
C25	0.0422 (15)	0.0280 (12)	0.0367 (14)	0.0103 (10)	−0.0029 (11)	−0.0034 (10)
C26	0.067 (2)	0.0246 (12)	0.0316 (13)	0.0007 (12)	0.0102 (13)	0.0041 (10)
C27	0.0556 (17)	0.0241 (11)	0.0363 (13)	−0.0096 (10)	0.0244 (13)	−0.0013 (9)
C27A	0.0263 (10)	0.0216 (9)	0.0283 (10)	−0.0020 (7)	0.0135 (8)	−0.0026 (7)
N27A	0.0263 (10)	0.0216 (9)	0.0283 (10)	−0.0020 (7)	0.0135 (8)	−0.0026 (7)
Zn1	0.01984 (12)	0.02715 (13)	0.02316 (13)	0.00151 (9)	0.00564 (9)	0.00513 (9)
Cl1	0.0206 (2)	0.0329 (3)	0.0284 (2)	0.00323 (18)	0.00568 (19)	0.0096 (2)
Cl2	0.01846 (15)	0.02384 (12)	0.03012 (12)	0.00196 (10)	0.01175 (9)	0.01045 (8)
I2	0.01846 (15)	0.02384 (12)	0.03012 (12)	0.00196 (10)	0.01175 (9)	0.01045 (8)
Cl3	0.0298 (3)	0.0181 (4)	0.0504 (5)	0.0077 (4)	0.0154 (3)	0.0078 (4)
I3	0.0298 (3)	0.0181 (4)	0.0504 (5)	0.0077 (4)	0.0154 (3)	0.0078 (4)
Cl4	0.0276 (3)	0.0452 (4)	0.0222 (4)	−0.0028 (2)	0.0058 (4)	−0.0034 (4)
I4	0.0276 (3)	0.0452 (4)	0.0222 (4)	−0.0028 (2)	0.0058 (4)	−0.0034 (4)

### Bis(2-methylimidazo[1,5-a]pyridinium) trichloridoiodidozincate(II), (I) Geometric parameters (Å, º)

**Table:**

C11—N17A	1.363 (3)	N22—C23	1.332 (3)
C11—C17A	1.363 (3)	N22—C22	1.465 (3)
C11—N12	1.364 (3)	C22—H22A	0.9800
C11—H11	0.9500	C22—H22B	0.9800
N12—C13	1.338 (3)	C22—H22C	0.9800
N12—C12	1.462 (3)	C23—C23A	1.338 (3)
C12—H12A	0.9800	C23—N23A	1.338 (3)
C12—H12B	0.9800	C23—H23	0.9500
C12—H12C	0.9800	N23A—C27A	1.400 (3)
C13—C13A	1.341 (3)	N23A—C24	1.401 (3)
C13—N13A	1.341 (3)	C23A—N27A	1.400 (3)
C13—H13	0.9500	C23A—C24	1.401 (3)
N13A—C14	1.392 (3)	C24—C25	1.348 (4)
N13A—C17A	1.408 (3)	C24—H24	0.9500
C13A—C14	1.392 (3)	C25—C26	1.406 (5)
C13A—N17A	1.408 (3)	C25—H25	0.9500
C14—C15	1.345 (4)	C26—C27	1.347 (5)
C14—H14	0.9500	C26—H26	0.9500
C15—C16	1.432 (4)	C27—N27A	1.422 (4)
C15—H15	0.9500	C27—C27A	1.422 (4)
C16—C17	1.350 (4)	C27—H27	0.9500
C16—H16	0.9500	Zn1—Cl3	2.2689 (10)
C17—N17A	1.411 (3)	Zn1—Cl4	2.2780 (11)
C17—C17A	1.411 (3)	Zn1—Cl1	2.2884 (6)
C17—H17	0.9500	Zn1—Cl2	2.346 (3)
C21—N27A	1.365 (4)	Zn1—I3	2.542 (4)
C21—C27A	1.365 (4)	Zn1—I4	2.568 (3)
C21—N22	1.368 (4)	Zn1—I2	2.5969 (4)
C21—H21	0.9500		
			
N17A—C11—N12	107.2 (2)	H22A—C22—H22C	109.5
C17A—C11—N12	107.2 (2)	H22B—C22—H22C	109.5
C17A—C11—H11	126.4	N22—C23—C23A	107.6 (2)
N12—C11—H11	126.4	N22—C23—N23A	107.6 (2)
C13—N12—C11	110.52 (19)	N22—C23—H23	126.2
C13—N12—C12	125.2 (2)	N23A—C23—H23	126.2
C11—N12—C12	124.3 (2)	C23—N23A—C27A	109.2 (2)
N12—C12—H12A	109.5	C23—N23A—C24	129.8 (2)
N12—C12—H12B	109.5	C27A—N23A—C24	121.1 (2)
H12A—C12—H12B	109.5	C23—C23A—N27A	109.2 (2)
N12—C12—H12C	109.5	C23—C23A—C24	129.8 (2)
H12A—C12—H12C	109.5	N27A—C23A—C24	121.1 (2)
H12B—C12—H12C	109.5	C25—C24—N23A	118.2 (3)
N12—C13—C13A	107.4 (2)	C25—C24—C23A	118.2 (3)
N12—C13—N13A	107.4 (2)	C25—C24—H24	120.9
N12—C13—H13	126.3	N23A—C24—H24	120.9
N13A—C13—H13	126.3	C24—C25—C26	121.6 (3)
C13—N13A—C14	129.9 (2)	C24—C25—H25	119.2
C13—N13A—C17A	108.77 (19)	C26—C25—H25	119.2
C14—N13A—C17A	121.32 (19)	C27—C26—C25	121.4 (3)
C13—C13A—C14	129.9 (2)	C27—C26—H26	119.3
C13—C13A—N17A	108.77 (19)	C25—C26—H26	119.3
C14—C13A—N17A	121.32 (19)	C26—C27—N27A	118.6 (3)
C15—C14—N13A	118.6 (2)	C26—C27—C27A	118.6 (3)
C15—C14—C13A	118.6 (2)	C26—C27—H27	120.7
C15—C14—H14	120.7	C27A—C27—H27	120.7
N13A—C14—H14	120.7	C21—C27A—N23A	105.9 (2)
C14—C15—C16	120.9 (2)	C21—C27A—C27	135.0 (2)
C14—C15—H15	119.5	N23A—C27A—C27	119.1 (2)
C16—C15—H15	119.5	C21—N27A—C23A	105.9 (2)
C17—C16—C15	121.3 (2)	C21—N27A—C27	135.0 (2)
C17—C16—H16	119.4	C23A—N27A—C27	119.1 (2)
C15—C16—H16	119.4	Cl3—Zn1—Cl4	112.60 (5)
C16—C17—N17A	118.5 (2)	Cl3—Zn1—Cl1	108.40 (4)
C16—C17—C17A	118.5 (2)	Cl4—Zn1—Cl1	107.71 (4)
C16—C17—H17	120.7	Cl3—Zn1—Cl2	110.84 (13)
C17A—C17—H17	120.7	Cl4—Zn1—Cl2	106.83 (14)
C11—C17A—N13A	106.19 (18)	Cl1—Zn1—Cl2	110.41 (12)
C11—C17A—C17	134.5 (2)	Cl3—Zn1—I3	3.2 (2)
N13A—C17A—C17	119.4 (2)	Cl4—Zn1—I3	115.8 (2)
C11—N17A—C13A	106.19 (18)	Cl1—Zn1—I3	106.36 (19)
C11—N17A—C17	134.5 (2)	Cl2—Zn1—I3	109.7 (2)
C13A—N17A—C17	119.4 (2)	Cl3—Zn1—I4	107.23 (14)
N27A—C21—N22	107.2 (2)	Cl4—Zn1—I4	5.44 (15)
C27A—C21—N22	107.2 (2)	Cl1—Zn1—I4	109.51 (13)
C27A—C21—H21	126.4	Cl2—Zn1—I4	110.37 (19)
N22—C21—H21	126.4	I3—Zn1—I4	110.4 (2)
C23—N22—C21	110.1 (2)	Cl3—Zn1—I2	109.83 (4)
C23—N22—C22	124.1 (3)	Cl4—Zn1—I2	106.78 (4)
C21—N22—C22	125.7 (3)	Cl1—Zn1—I2	111.54 (2)
N22—C22—H22A	109.5	Cl2—Zn1—I2	1.24 (13)
N22—C22—H22B	109.5	I3—Zn1—I2	108.8 (2)
H22A—C22—H22B	109.5	I4—Zn1—I2	110.22 (13)
N22—C22—H22C	109.5		
			
N17A—C11—N12—C13	0.4 (3)	N27A—C21—N22—C23	0.0 (3)
C17A—C11—N12—C13	0.4 (3)	C27A—C21—N22—C23	0.0 (3)
N17A—C11—N12—C12	179.7 (2)	N27A—C21—N22—C22	−177.8 (2)
C17A—C11—N12—C12	179.7 (2)	C27A—C21—N22—C22	−177.8 (2)
C11—N12—C13—C13A	−0.6 (3)	C21—N22—C23—C23A	0.0 (3)
C12—N12—C13—C13A	−179.8 (2)	C22—N22—C23—C23A	177.8 (2)
C11—N12—C13—N13A	−0.6 (3)	C21—N22—C23—N23A	0.0 (3)
C12—N12—C13—N13A	−179.8 (2)	C22—N22—C23—N23A	177.8 (2)
N12—C13—N13A—C14	−177.5 (2)	N22—C23—N23A—C27A	0.1 (3)
N12—C13—N13A—C17A	0.4 (3)	N22—C23—N23A—C24	−179.5 (2)
N12—C13—C13A—C14	−177.5 (2)	N22—C23—C23A—N27A	0.1 (3)
N12—C13—C13A—N17A	0.4 (3)	N22—C23—C23A—C24	−179.5 (2)
C13—N13A—C14—C15	177.2 (2)	C23—N23A—C24—C25	179.6 (2)
C17A—N13A—C14—C15	−0.5 (3)	C27A—N23A—C24—C25	0.2 (3)
C13—C13A—C14—C15	177.2 (2)	C23—C23A—C24—C25	179.6 (2)
N17A—C13A—C14—C15	−0.5 (3)	N27A—C23A—C24—C25	0.2 (3)
N13A—C14—C15—C16	1.6 (4)	N23A—C24—C25—C26	−0.8 (4)
C13A—C14—C15—C16	1.6 (4)	C23A—C24—C25—C26	−0.8 (4)
C14—C15—C16—C17	−1.0 (4)	C24—C25—C26—C27	0.8 (4)
C15—C16—C17—N17A	−0.8 (4)	C25—C26—C27—N27A	−0.1 (4)
C15—C16—C17—C17A	−0.8 (4)	C25—C26—C27—C27A	−0.1 (4)
N12—C11—C17A—N13A	−0.1 (2)	N22—C21—C27A—N23A	0.1 (2)
N12—C11—C17A—C17	178.9 (2)	N22—C21—C27A—C27	178.8 (3)
C13—N13A—C17A—C11	−0.2 (2)	C23—N23A—C27A—C21	−0.1 (2)
C14—N13A—C17A—C11	177.9 (2)	C24—N23A—C27A—C21	179.5 (2)
C13—N13A—C17A—C17	−179.4 (2)	C23—N23A—C27A—C27	−179.1 (2)
C14—N13A—C17A—C17	−1.3 (3)	C24—N23A—C27A—C27	0.5 (3)
C16—C17—C17A—C11	−177.1 (2)	C26—C27—C27A—C21	−179.1 (3)
C16—C17—C17A—N13A	1.9 (3)	C26—C27—C27A—N23A	−0.5 (3)
N12—C11—N17A—C13A	−0.1 (2)	N22—C21—N27A—C23A	0.1 (2)
N12—C11—N17A—C17	178.9 (2)	N22—C21—N27A—C27	178.8 (3)
C13—C13A—N17A—C11	−0.2 (2)	C23—C23A—N27A—C21	−0.1 (2)
C14—C13A—N17A—C11	177.9 (2)	C24—C23A—N27A—C21	179.5 (2)
C13—C13A—N17A—C17	−179.4 (2)	C23—C23A—N27A—C27	−179.1 (2)
C14—C13A—N17A—C17	−1.3 (3)	C24—C23A—N27A—C27	0.5 (3)
C16—C17—N17A—C11	−177.1 (2)	C26—C27—N27A—C21	−179.1 (3)
C16—C17—N17A—C13A	1.9 (3)	C26—C27—N27A—C23A	−0.5 (3)

### Bis(2-methylimidazo[1,5-a]pyridinium) trichloridoiodidozincate(II), (I) Hydrogen-bond geometry (Å, º)

**Table:**

*D*—H···*A*	*D*—H	H···*A*	*D*···*A*	*D*—H···*A*
C13—H13···I4^i^	0.95	2.88	3.349 (5)	112
C12—H12*B*···Cl4^i^	0.98	2.78	3.562 (3)	137
C12—H12*C*···Cl3^ii^	0.98	2.80	3.698 (3)	153
C11—H11···Cl1^iii^	0.95	2.72	3.484 (2)	138
C22—H22*B*···I2^iii^	0.98	3.06	3.946 (3)	151
C22—H22*C*···I3^iii^	0.98	3.00	3.562 (9)	117
C23—H23···Cl1^ii^	0.95	2.83	3.486 (3)	127
C24—H24···Cl3^ii^	0.95	2.71	3.579 (3)	152
C27—H27···Cl4^iv^	0.95	2.75	3.624 (3)	153

Symmetry codes: (i) −*x*+2, −*y*+1, −*z*+1; (ii) *x*, *y*+1, *z*; (iii) −*x*+1, −*y*+1, −*z*; (iv) −*x*+1, −*y*+1, −*z*+1.

### Bis(2-methylimidazo[1,5-a]pyridinium) dibromidodichloridozincate(II) (II) Crystal data

**Table:**

(C_8_H_9_N_2_)_2_[CdBr_2.42_Cl_1.58_]	*Z* = 2
*M**_r_* = 628.14	*F*(000) = 603
Triclinic, *P*1	*D*_x_ = 2.003 Mg m^−^^3^
*a* = 9.5172 (5) Å	Mo *K*α radiation, λ = 0.71073 Å
*b* = 10.8293 (6) Å	Cell parameters from 6082 reflections
*c* = 10.9697 (6) Å	θ = 2.5–31.9°
α = 99.620 (5)°	µ = 5.90 mm^−^^1^
β = 110.413 (5)°	*T* = 100 K
γ = 90.827 (5)°	Plate, colourless
*V* = 1041.45 (10) Å^3^	0.36 × 0.28 × 0.11 mm

### Bis(2-methylimidazo[1,5-a]pyridinium) dibromidodichloridozincate(II) (II) Data collection

**Table:**

Oxford Diffraction Gemini diffractometer	6879 independent reflections
Graphite monochromator	5371 reflections with *I* > 2σ(*I*)
Detector resolution: 10.4738 pixels mm^-1^	*R*_int_ = 0.036
ω scans	θ_max_ = 32.7°, θ_min_ = 2.0°
Absorption correction: analytical CrysAlis Pro (Rigaku OD, 2016)	*h* = −13→14
*T*_min_ = 0.206, *T*_max_ = 0.53	*k* = −16→16
15893 measured reflections	*l* = −15→16

### Bis(2-methylimidazo[1,5-a]pyridinium) dibromidodichloridozincate(II) (II) Refinement

**Table:**

Refinement on *F*^2^	8 restraints
Least-squares matrix: full	Hydrogen site location: inferred from neighbouring sites
*R*[*F*^2^ > 2σ(*F*^2^)] = 0.036	H-atom parameters constrained
*wR*(*F*^2^) = 0.068	*w* = 1/[σ^2^(*F*_o_^2^) + (0.019*P*)^2^ + 0.3916*P*] where *P* = (*F*_o_^2^ + 2*F*_c_^2^)/3
*S* = 1.04	(Δ/σ)_max_ = 0.001
6879 reflections	Δρ_max_ = 0.89 e Å^−^^3^
246 parameters	Δρ_min_ = −0.77 e Å^−^^3^

### Bis(2-methylimidazo[1,5-a]pyridinium) dibromidodichloridozincate(II) (II) Special details

**Table:**

Geometry. All esds (except the esd in the dihedral angle between two l.s. planes) are estimated using the full covariance matrix. The cell esds are taken into account individually in the estimation of esds in distances, angles and torsion angles; correlations between esds in cell parameters are only used when they are defined by crystal symmetry. An approximate (isotropic) treatment of cell esds is used for estimating esds involving l.s. planes.
Refinement. The halide atom sites were modelled as being part Br and part Cl with site occupancies refined to 0.417 (2), 0.857 (2), 0.558 (2) and 0.590 (2) for the Br occupancy for sites 1-4 with the Cl occupancies being the complements. Cd-X bond lengths of the disordered atoms were restrained to ideal values. The cations were modelled as being rotationally disordered by 180 degrees. The site occupancies refined to 0.73 (2) and its complement for cation 1 and 0.75 (2) and its complement for cation 2. Three reflections with very poor agreement were omitted from the refinement.

### Bis(2-methylimidazo[1,5-a]pyridinium) dibromidodichloridozincate(II) (II) Fractional atomic coordinates and isotropic or equivalent isotropic displacement parameters (Å^2^)

**Table:**

	*x*	*y*	*z*	*U*_iso_*/*U*_eq_	Occ. (<1)
C11	0.6551 (3)	0.6498 (2)	0.1036 (3)	0.0215 (6)	
H11	0.643060	0.698431	0.036699	0.026*	
N12	0.7851 (3)	0.6065 (2)	0.1763 (2)	0.0203 (5)	
C12	0.9297 (3)	0.6271 (3)	0.1610 (3)	0.0269 (7)	
H12A	0.988955	0.697944	0.228531	0.040*	
H12B	0.912731	0.645988	0.072888	0.040*	
H12C	0.984506	0.551360	0.171451	0.040*	
C13	0.7613 (3)	0.5408 (2)	0.2620 (3)	0.0208 (6)	
H13	0.834623	0.500985	0.323175	0.025*	
N13A	0.6145 (3)	0.5421 (2)	0.2452 (3)	0.0199 (6)	0.73 (2)
C13A	0.6145 (3)	0.5421 (2)	0.2452 (3)	0.0199 (6)	0.27 (2)
C14	0.5319 (4)	0.4908 (3)	0.3114 (3)	0.0274 (7)	
H14	0.579079	0.444386	0.378932	0.033*	
C15	0.3853 (4)	0.5089 (3)	0.2773 (4)	0.0329 (8)	
H15	0.328458	0.476488	0.322639	0.039*	
C16	0.3130 (4)	0.5764 (3)	0.1734 (4)	0.0313 (7)	
H16	0.208509	0.586480	0.150273	0.038*	
C17	0.3887 (3)	0.6259 (2)	0.1076 (3)	0.0243 (6)	
H17	0.339129	0.669834	0.038431	0.029*	
C17A	0.5446 (3)	0.6103 (2)	0.1446 (3)	0.0197 (6)	0.73 (2)
N17A	0.5446 (3)	0.6103 (2)	0.1446 (3)	0.0197 (6)	0.27 (2)
C21	0.3022 (3)	0.8993 (2)	0.3242 (3)	0.0221 (6)	
H21	0.207038	0.901456	0.334175	0.027*	
N22	0.3367 (3)	0.9351 (2)	0.2233 (2)	0.0219 (5)	
C22	0.2316 (4)	0.9859 (3)	0.1121 (3)	0.0309 (7)	
H22A	0.285214	1.008631	0.056212	0.046*	
H22B	0.190771	1.060694	0.146702	0.046*	
H22C	0.149190	0.922204	0.059430	0.046*	
C23	0.4808 (4)	0.9189 (2)	0.2403 (3)	0.0225 (6)	
H23	0.531495	0.936507	0.183524	0.027*	
N23A	0.5413 (3)	0.8728 (2)	0.3535 (2)	0.0181 (5)	0.75 (2)
C23A	0.5413 (3)	0.8728 (2)	0.3535 (2)	0.0181 (5)	0.25 (2)
C24	0.6873 (3)	0.8389 (3)	0.4152 (3)	0.0250 (6)	
H24	0.762633	0.847636	0.378149	0.030*	
C25	0.7187 (4)	0.7935 (3)	0.5287 (3)	0.0285 (7)	
H25	0.816635	0.768429	0.570170	0.034*	
C26	0.6081 (4)	0.7824 (3)	0.5877 (3)	0.0285 (7)	
H26	0.634114	0.752055	0.668515	0.034*	
C27	0.4670 (4)	0.8146 (2)	0.5297 (3)	0.0252 (7)	
H27	0.393603	0.807294	0.569120	0.030*	
C27A	0.4298 (3)	0.8598 (2)	0.4078 (3)	0.0211 (6)	0.75 (2)
N27A	0.4298 (3)	0.8598 (2)	0.4078 (3)	0.0211 (6)	0.25 (2)
Cd1	0.84620 (2)	0.18609 (2)	0.25086 (2)	0.01928 (6)	
Br1	0.5610 (3)	0.1881 (11)	0.1247 (10)	0.0222 (4)	0.417 (2)
Cl1	0.5727 (5)	0.1844 (19)	0.1340 (18)	0.0222 (4)	0.583 (2)
Br2	1.0021 (2)	0.2985 (2)	0.1431 (2)	0.02319 (16)	0.857 (2)
Cl2	1.003 (3)	0.291 (3)	0.155 (3)	0.02319 (16)	0.143 (2)
Br3	0.9014 (7)	−0.04313 (18)	0.2368 (6)	0.0262 (2)	0.558 (2)
Cl3	0.902 (2)	−0.0282 (6)	0.2367 (19)	0.0262 (2)	0.442 (2)
Br4	0.8962 (4)	0.31677 (16)	0.48575 (14)	0.0245 (3)	0.590 (2)
Cl4	0.8977 (14)	0.2952 (7)	0.4831 (5)	0.0245 (3)	0.410 (2)

### Bis(2-methylimidazo[1,5-a]pyridinium) dibromidodichloridozincate(II) (II) Atomic displacement parameters (Å^2^)

**Table:**

	*U*^11^	*U*^22^	*U*^33^	*U*^12^	*U*^13^	*U*^23^
C11	0.0217 (16)	0.0175 (13)	0.0258 (15)	0.0023 (10)	0.0095 (13)	0.0031 (11)
N12	0.0165 (12)	0.0187 (11)	0.0269 (13)	0.0025 (9)	0.0101 (11)	0.0026 (9)
C12	0.0190 (16)	0.0296 (16)	0.0397 (18)	0.0051 (12)	0.0179 (14)	0.0103 (13)
C13	0.0176 (15)	0.0177 (13)	0.0269 (15)	0.0012 (10)	0.0082 (12)	0.0030 (10)
N13A	0.0163 (13)	0.0165 (12)	0.0273 (14)	0.0012 (9)	0.0098 (11)	0.0011 (9)
C13A	0.0163 (13)	0.0165 (12)	0.0273 (14)	0.0012 (9)	0.0098 (11)	0.0011 (9)
C14	0.0330 (19)	0.0191 (14)	0.0350 (17)	0.0005 (12)	0.0183 (15)	0.0048 (12)
C15	0.035 (2)	0.0243 (15)	0.048 (2)	−0.0025 (13)	0.0285 (18)	0.0015 (14)
C16	0.0151 (15)	0.0263 (15)	0.050 (2)	−0.0026 (11)	0.0148 (15)	−0.0063 (14)
C17	0.0172 (15)	0.0210 (14)	0.0313 (16)	0.0025 (11)	0.0077 (13)	−0.0027 (11)
C17A	0.0176 (14)	0.0143 (12)	0.0251 (14)	0.0008 (9)	0.0075 (12)	−0.0020 (10)
N17A	0.0176 (14)	0.0143 (12)	0.0251 (14)	0.0008 (9)	0.0075 (12)	−0.0020 (10)
C21	0.0206 (15)	0.0199 (13)	0.0246 (15)	0.0023 (11)	0.0090 (13)	−0.0012 (11)
N22	0.0241 (14)	0.0179 (11)	0.0205 (12)	0.0026 (9)	0.0054 (11)	0.0007 (9)
C22	0.0335 (19)	0.0267 (16)	0.0252 (16)	0.0060 (13)	0.0020 (15)	0.0037 (12)
C23	0.0291 (17)	0.0176 (13)	0.0218 (14)	0.0028 (11)	0.0114 (13)	0.0013 (10)
N23A	0.0214 (14)	0.0159 (11)	0.0189 (12)	0.0026 (9)	0.0104 (11)	0.0012 (9)
C23A	0.0214 (14)	0.0159 (11)	0.0189 (12)	0.0026 (9)	0.0104 (11)	0.0012 (9)
C24	0.0182 (15)	0.0247 (15)	0.0331 (17)	0.0007 (11)	0.0139 (14)	−0.0027 (12)
C25	0.0250 (17)	0.0191 (14)	0.0332 (17)	0.0028 (11)	0.0023 (14)	0.0000 (12)
C26	0.038 (2)	0.0200 (14)	0.0233 (15)	−0.0037 (13)	0.0059 (14)	0.0050 (11)
C27	0.0344 (19)	0.0206 (14)	0.0241 (15)	−0.0060 (12)	0.0171 (14)	−0.0004 (11)
C27A	0.0240 (15)	0.0159 (13)	0.0251 (14)	−0.0011 (10)	0.0137 (12)	−0.0025 (10)
N27A	0.0240 (15)	0.0159 (13)	0.0251 (14)	−0.0011 (10)	0.0137 (12)	−0.0025 (10)
Cd1	0.01779 (11)	0.02089 (11)	0.01967 (10)	0.00058 (7)	0.00668 (8)	0.00518 (7)
Br1	0.0163 (4)	0.0287 (6)	0.0213 (13)	0.0048 (8)	0.0034 (6)	0.0104 (7)
Cl1	0.0163 (4)	0.0287 (6)	0.0213 (13)	0.0048 (8)	0.0034 (6)	0.0104 (7)
Br2	0.02510 (19)	0.0221 (4)	0.0273 (5)	0.00083 (18)	0.0134 (3)	0.0089 (2)
Cl2	0.02510 (19)	0.0221 (4)	0.0273 (5)	0.00083 (18)	0.0134 (3)	0.0089 (2)
Br3	0.0274 (3)	0.0170 (6)	0.0415 (3)	0.0068 (8)	0.0173 (2)	0.0138 (8)
Cl3	0.0274 (3)	0.0170 (6)	0.0415 (3)	0.0068 (8)	0.0173 (2)	0.0138 (8)
Br4	0.0239 (2)	0.0248 (8)	0.0214 (2)	−0.0037 (6)	0.00643 (18)	−0.0006 (3)
Cl4	0.0239 (2)	0.0248 (8)	0.0214 (2)	−0.0037 (6)	0.00643 (18)	−0.0006 (3)

### Bis(2-methylimidazo[1,5-a]pyridinium) dibromidodichloridozincate(II) (II) Geometric parameters (Å, º)

**Table:**

C11—N12	1.356 (4)	N22—C23	1.337 (4)
C11—N17A	1.368 (4)	N22—C22	1.477 (4)
C11—C17A	1.368 (4)	C22—H22A	0.9800
C11—H11	0.9500	C22—H22B	0.9800
N12—C13	1.345 (4)	C22—H22C	0.9800
N12—C12	1.462 (3)	C23—C23A	1.357 (4)
C12—H12A	0.9800	C23—N23A	1.357 (4)
C12—H12B	0.9800	C23—H23	0.9500
C12—H12C	0.9800	N23A—C24	1.402 (4)
C13—C13A	1.345 (4)	N23A—C27A	1.402 (3)
C13—N13A	1.345 (4)	C23A—C24	1.402 (4)
C13—H13	0.9500	C23A—N27A	1.402 (3)
N13A—C14	1.404 (4)	C24—C25	1.353 (4)
N13A—C17A	1.408 (4)	C24—H24	0.9500
C13A—C14	1.404 (4)	C25—C26	1.427 (4)
C13A—N17A	1.408 (4)	C25—H25	0.9500
C14—C15	1.339 (4)	C26—C27	1.350 (4)
C14—H14	0.9500	C26—H26	0.9500
C15—C16	1.433 (5)	C27—N27A	1.431 (4)
C15—H15	0.9500	C27—C27A	1.431 (4)
C16—C17	1.344 (4)	C27—H27	0.9500
C16—H16	0.9500	Cd1—Cl3	2.380 (4)
C17—N17A	1.415 (4)	Cd1—Cl2	2.460 (5)
C17—C17A	1.415 (4)	Cd1—Cl1	2.467 (3)
C17—H17	0.9500	Cd1—Cl4	2.497 (4)
C21—N27A	1.366 (4)	Cd1—Br3	2.5353 (12)
C21—C27A	1.366 (4)	Cd1—Br1	2.5834 (17)
C21—N22	1.369 (4)	Cd1—Br2	2.5950 (5)
C21—H21	0.9500	Cd1—Br4	2.6029 (11)
			
N12—C11—N17A	107.3 (2)	N22—C23—N23A	107.3 (2)
N12—C11—C17A	107.3 (2)	N22—C23—H23	126.3
N12—C11—H11	126.3	N23A—C23—H23	126.3
C17A—C11—H11	126.3	C23—N23A—C24	130.4 (3)
C13—N12—C11	110.5 (2)	C23—N23A—C27A	108.7 (3)
C13—N12—C12	125.2 (3)	C24—N23A—C27A	120.9 (2)
C11—N12—C12	124.3 (2)	C23—C23A—C24	130.4 (3)
N12—C12—H12A	109.5	C23—C23A—N27A	108.7 (3)
N12—C12—H12B	109.5	C24—C23A—N27A	120.9 (2)
H12A—C12—H12B	109.5	C25—C24—N23A	118.6 (3)
N12—C12—H12C	109.5	C25—C24—C23A	118.6 (3)
H12A—C12—H12C	109.5	C25—C24—H24	120.7
H12B—C12—H12C	109.5	N23A—C24—H24	120.7
C13A—C13—N12	107.4 (3)	C24—C25—C26	121.6 (3)
N13A—C13—N12	107.4 (3)	C24—C25—H25	119.2
N13A—C13—H13	126.3	C26—C25—H25	119.2
N12—C13—H13	126.3	C27—C26—C25	120.6 (3)
C13—N13A—C14	130.7 (3)	C27—C26—H26	119.7
C13—N13A—C17A	108.6 (2)	C25—C26—H26	119.7
C14—N13A—C17A	120.8 (2)	C26—C27—N27A	118.9 (3)
C13—C13A—C14	130.7 (3)	C26—C27—C27A	118.9 (3)
C13—C13A—N17A	108.6 (2)	C26—C27—H27	120.5
C14—C13A—N17A	120.8 (2)	C27A—C27—H27	120.5
C15—C14—N13A	118.6 (3)	C21—C27A—N23A	106.2 (2)
C15—C14—C13A	118.6 (3)	C21—C27A—C27	134.4 (3)
C15—C14—H14	120.7	N23A—C27A—C27	119.4 (3)
N13A—C14—H14	120.7	C21—N27A—C23A	106.2 (2)
C14—C15—C16	120.8 (3)	C21—N27A—C27	134.4 (3)
C14—C15—H15	119.6	C23A—N27A—C27	119.4 (3)
C16—C15—H15	119.6	Cl3—Cd1—Cl2	107.3 (10)
C17—C16—C15	122.0 (3)	Cl3—Cd1—Cl1	106.1 (7)
C17—C16—H16	119.0	Cl2—Cd1—Cl1	114.9 (9)
C15—C16—H16	119.0	Cl3—Cd1—Cl4	112.7 (5)
C16—C17—N17A	117.9 (3)	Cl2—Cd1—Cl4	109.6 (9)
C16—C17—C17A	117.9 (3)	Cl1—Cd1—Cl4	106.3 (6)
C16—C17—H17	121.1	Cl3—Cd1—Br3	1.0 (6)
C17A—C17—H17	121.1	Cl2—Cd1—Br3	108.4 (9)
C11—C17A—N13A	106.3 (2)	Cl1—Cd1—Br3	105.3 (5)
C11—C17A—C17	133.9 (3)	Cl4—Cd1—Br3	112.4 (2)
N13A—C17A—C17	119.9 (2)	Cl3—Cd1—Br1	107.0 (5)
C11—N17A—C13A	106.3 (2)	Cl2—Cd1—Br1	113.4 (8)
C11—N17A—C17	133.9 (3)	Cl1—Cd1—Br1	1.5 (7)
C13A—N17A—C17	119.9 (2)	Cl4—Cd1—Br1	106.9 (4)
N27A—C21—N22	107.5 (3)	Br3—Cd1—Br1	106.2 (3)
C27A—C21—N22	107.5 (3)	Cl3—Cd1—Br2	108.3 (5)
C27A—C21—H21	126.2	Cl2—Cd1—Br2	2.1 (8)
N22—C21—H21	126.2	Cl1—Cd1—Br2	112.8 (5)
C23—N22—C21	110.2 (2)	Cl4—Cd1—Br2	110.6 (2)
C23—N22—C22	124.3 (3)	Br3—Cd1—Br2	109.34 (14)
C21—N22—C22	125.5 (3)	Br1—Cd1—Br2	111.3 (3)
N22—C22—H22A	109.5	Cl3—Cd1—Br4	117.3 (5)
N22—C22—H22B	109.5	Cl2—Cd1—Br4	106.6 (9)
H22A—C22—H22B	109.5	Cl1—Cd1—Br4	104.9 (5)
N22—C22—H22C	109.5	Cl4—Cd1—Br4	4.6 (2)
H22A—C22—H22C	109.5	Br3—Cd1—Br4	117.00 (15)
H22B—C22—H22C	109.5	Br1—Cd1—Br4	105.4 (3)
N22—C23—C23A	107.3 (2)	Br2—Cd1—Br4	107.57 (8)
			
N17A—C11—N12—C13	0.0 (3)	N27A—C21—N22—C23	0.3 (3)
C17A—C11—N12—C13	0.0 (3)	C27A—C21—N22—C23	0.3 (3)
N17A—C11—N12—C12	179.1 (2)	N27A—C21—N22—C22	−179.0 (2)
C17A—C11—N12—C12	179.1 (2)	C27A—C21—N22—C22	−179.0 (2)
C11—N12—C13—C13A	−0.3 (3)	C21—N22—C23—C23A	−0.3 (3)
C12—N12—C13—C13A	−179.3 (2)	C22—N22—C23—C23A	179.0 (2)
C11—N12—C13—N13A	−0.3 (3)	C21—N22—C23—N23A	−0.3 (3)
C12—N12—C13—N13A	−179.3 (2)	C22—N22—C23—N23A	179.0 (2)
N12—C13—N13A—C14	−178.2 (3)	N22—C23—N23A—C24	179.5 (2)
N12—C13—N13A—C17A	0.4 (3)	N22—C23—N23A—C27A	0.2 (3)
N12—C13—C13A—C14	−178.2 (3)	N22—C23—C23A—C24	179.5 (2)
N12—C13—C13A—N17A	0.4 (3)	N22—C23—C23A—N27A	0.2 (3)
C13—N13A—C14—C15	178.1 (3)	C23—N23A—C24—C25	−179.4 (3)
C17A—N13A—C14—C15	−0.4 (4)	C27A—N23A—C24—C25	−0.1 (4)
C13—C13A—C14—C15	178.1 (3)	C23—C23A—C24—C25	−179.4 (3)
N17A—C13A—C14—C15	−0.4 (4)	N27A—C23A—C24—C25	−0.1 (4)
N13A—C14—C15—C16	1.5 (4)	N23A—C24—C25—C26	−1.5 (4)
C13A—C14—C15—C16	1.5 (4)	C23A—C24—C25—C26	−1.5 (4)
C14—C15—C16—C17	−1.1 (5)	C24—C25—C26—C27	1.5 (4)
C15—C16—C17—N17A	−0.5 (4)	C25—C26—C27—N27A	0.1 (4)
C15—C16—C17—C17A	−0.5 (4)	C25—C26—C27—C27A	0.1 (4)
N12—C11—C17A—N13A	0.2 (3)	N22—C21—C27A—N23A	−0.2 (3)
N12—C11—C17A—C17	179.7 (3)	N22—C21—C27A—C27	178.5 (3)
C13—N13A—C17A—C11	−0.4 (3)	C23—N23A—C27A—C21	0.0 (3)
C14—N13A—C17A—C11	178.3 (2)	C24—N23A—C27A—C21	−179.4 (2)
C13—N13A—C17A—C17	−179.9 (2)	C23—N23A—C27A—C27	−178.9 (2)
C14—N13A—C17A—C17	−1.2 (4)	C24—N23A—C27A—C27	1.7 (4)
C16—C17—C17A—C11	−177.8 (3)	C26—C27—C27A—C21	179.8 (3)
C16—C17—C17A—N13A	1.6 (4)	C26—C27—C27A—N23A	−1.7 (4)
N12—C11—N17A—C13A	0.2 (3)	N22—C21—N27A—C23A	−0.2 (3)
N12—C11—N17A—C17	179.7 (3)	N22—C21—N27A—C27	178.5 (3)
C13—C13A—N17A—C11	−0.4 (3)	C23—C23A—N27A—C21	0.0 (3)
C14—C13A—N17A—C11	178.3 (2)	C24—C23A—N27A—C21	−179.4 (2)
C13—C13A—N17A—C17	−179.9 (2)	C23—C23A—N27A—C27	−178.9 (2)
C14—C13A—N17A—C17	−1.2 (4)	C24—C23A—N27A—C27	1.7 (4)
C16—C17—N17A—C11	−177.8 (3)	C26—C27—N27A—C21	179.8 (3)
C16—C17—N17A—C13A	1.6 (4)	C26—C27—N27A—C23A	−1.7 (4)

### Bis(2-methylimidazo[1,5-a]pyridinium) dibromidodichloridozincate(II) (II) Hydrogen-bond geometry (Å, º)

**Table:**

*D*—H···*A*	*D*—H	H···*A*	*D*···*A*	*D*—H···*A*
C11—H11···Br1^i^	0.95	2.61	3.401 (9)	141
C12—H12*B*···Br2^ii^	0.98	2.90	3.820 (3)	156
C12—H12*C*···Br2	0.98	2.72	3.621 (4)	153
C13—H13···Br4	0.95	2.83	3.666 (4)	148
C13—H13···Br4^iii^	0.95	3.09	3.577 (4)	113
C17—H17···Br1^i^	0.95	2.92	3.649 (12)	134
C21—H21···Br3^iv^	0.95	2.84	3.685 (6)	149
C23—H23···Br1^v^	0.95	2.93	3.541 (12)	123
C24—H24···Br3^v^	0.95	2.75	3.627 (6)	155
C27—H27···Br4^vi^	0.95	2.87	3.657 (4)	141

Symmetry codes: (i) −*x*+1, −*y*+1, −*z*; (ii) −*x*+2, −*y*+1, −*z*; (iii) −*x*+2, −*y*+1, −*z*+1; (iv) *x*−1, *y*+1, *z*; (v) *x*, *y*+1, *z*; (vi) −*x*+1, −*y*+1, −*z*+1.

### Bis(2-methylimidazo[1,5-a]pyridinium) trichloridoiodidozincate(II) (III) Crystal data

**Table:**

(C_8_H_9_N_2_)_2_[CdCl_3.90_I_0.10_]	*Z* = 2
*M**_r_* = 529.69	*F*(000) = 523
Triclinic, *P*1	*D*_x_ = 1.745 Mg m^−^^3^
*a* = 9.4304 (3) Å	Cu *K*α radiation, λ = 1.54178 Å
*b* = 10.7968 (3) Å	Cell parameters from 10758 reflections
*c* = 10.7565 (3) Å	θ = 4.2–67.2°
α = 99.209 (3)°	µ = 14.69 mm^−^^1^
β = 110.746 (3)°	*T* = 100 K
γ = 90.837 (2)°	Needle, colourless
*V* = 1007.97 (5) Å^3^	0.25 × 0.08 × 0.04 mm

### Bis(2-methylimidazo[1,5-a]pyridinium) trichloridoiodidozincate(II) (III) Data collection

**Table:**

Oxford Diffraction Gemini diffractometer	3581 independent reflections
Radiation source: sealed X-ray tube, Enhance Ultra (Cu) X-ray Source	3309 reflections with *I* > 2σ(*I*)
Mirror monochromator	*R*_int_ = 0.041
Detector resolution: 10.4738 pixels mm^-1^	θ_max_ = 67.3°, θ_min_ = 4.2°
ω scans	*h* = −11→11
Absorption correction: analytical CrysAlis Pro (Rigaku OD, 2016)	*k* = −12→12
*T*_min_ = 0.052, *T*_max_ = 0.522	*l* = −12→12
18506 measured reflections	

### Bis(2-methylimidazo[1,5-a]pyridinium) trichloridoiodidozincate(II) (III) Refinement

**Table:**

Refinement on *F*^2^	2 restraints
Least-squares matrix: full	Hydrogen site location: inferred from neighbouring sites
*R*[*F*^2^ > 2σ(*F*^2^)] = 0.027	H-atom parameters constrained
*wR*(*F*^2^) = 0.068	*w* = 1/[σ^2^(*F*_o_^2^) + (0.0384*P*)^2^ + 0.6373*P*] where *P* = (*F*_o_^2^ + 2*F*_c_^2^)/3
*S* = 1.06	(Δ/σ)_max_ < 0.001
3581 reflections	Δρ_max_ = 0.79 e Å^−^^3^
234 parameters	Δρ_min_ = −0.46 e Å^−^^3^

### Bis(2-methylimidazo[1,5-a]pyridinium) trichloridoiodidozincate(II) (III) Special details

**Table:**

Geometry. All esds (except the esd in the dihedral angle between two l.s. planes) are estimated using the full covariance matrix. The cell esds are taken into account individually in the estimation of esds in distances, angles and torsion angles; correlations between esds in cell parameters are only used when they are defined by crystal symmetry. An approximate (isotropic) treatment of cell esds is used for estimating esds involving l.s. planes.
Refinement. The halogen site 2 was modelled as being part Cl and part I, with Cl site occupancies refined to 0.9008 (15) with the I site occupancies being its complement. Cd-X bond lengths of the disordered atoms were restrained to ideal values. The cations were modelled as being rotationally disordered by 180 degrees. The site occupancies refined to 0.72 (3) and its complement for cation 1 and 0.81 (3) and its complement for cation 2.

### Bis(2-methylimidazo[1,5-a]pyridinium) trichloridoiodidozincate(II) (III) Fractional atomic coordinates and isotropic or equivalent isotropic displacement parameters (Å^2^)

**Table:**

	*x*	*y*	*z*	*U*_iso_*/*U*_eq_	Occ. (<1)
C11	0.6607 (3)	0.6542 (3)	0.1045 (3)	0.0263 (6)	
H11	0.649443	0.704261	0.037015	0.032*	
N12	0.7924 (3)	0.6108 (2)	0.1810 (2)	0.0254 (5)	
C12	0.9396 (3)	0.6325 (3)	0.1681 (3)	0.0326 (7)	
H12A	1.010714	0.681187	0.252843	0.049*	
H12B	0.926939	0.679406	0.094506	0.049*	
H12C	0.979688	0.551513	0.148316	0.049*	
C13	0.7667 (3)	0.5435 (3)	0.2662 (3)	0.0279 (6)	
H13	0.840247	0.503253	0.329448	0.034*	
N13A	0.6172 (3)	0.5435 (2)	0.2456 (3)	0.0273 (7)	0.72 (3)
C13A	0.6172 (3)	0.5435 (2)	0.2456 (3)	0.0273 (7)	0.28 (3)
C14	0.5312 (4)	0.4912 (3)	0.3101 (3)	0.0371 (7)	
H14	0.577624	0.444977	0.380184	0.045*	
C15	0.3812 (4)	0.5080 (3)	0.2705 (4)	0.0409 (8)	
H15	0.321888	0.473974	0.314089	0.049*	
C16	0.3102 (4)	0.5760 (3)	0.1644 (4)	0.0367 (7)	
H16	0.204106	0.585364	0.137727	0.044*	
C17	0.3904 (3)	0.6272 (3)	0.1010 (3)	0.0303 (6)	
H17	0.342153	0.672136	0.030121	0.036*	
C17A	0.5482 (3)	0.6124 (3)	0.1428 (3)	0.0263 (7)	0.72 (3)
N17A	0.5482 (3)	0.6124 (3)	0.1428 (3)	0.0263 (7)	0.28 (3)
C21	0.2961 (3)	0.8984 (3)	0.3210 (3)	0.0300 (6)	
H21	0.200250	0.899221	0.331504	0.036*	
N22	0.3296 (3)	0.9360 (2)	0.2187 (2)	0.0307 (5)	
C22	0.2224 (4)	0.9883 (3)	0.1069 (3)	0.0408 (8)	
H22A	0.276822	1.018014	0.053509	0.061*	
H22B	0.175995	1.058734	0.143448	0.061*	
H22C	0.142796	0.922893	0.049311	0.061*	
C23	0.4755 (4)	0.9209 (3)	0.2355 (3)	0.0302 (6)	
H23	0.525500	0.939492	0.177260	0.036*	
N23A	0.5383 (3)	0.8745 (2)	0.3502 (2)	0.0271 (6)	0.81 (3)
C23A	0.5383 (3)	0.8745 (2)	0.3502 (2)	0.0271 (6)	0.19 (3)
C24	0.6866 (3)	0.8419 (3)	0.4123 (3)	0.0323 (7)	
H24	0.761877	0.851903	0.374101	0.039*	
C25	0.7201 (4)	0.7958 (3)	0.5286 (3)	0.0365 (7)	
H25	0.819879	0.771912	0.571491	0.044*	
C26	0.6093 (4)	0.7825 (3)	0.5874 (3)	0.0372 (7)	
H26	0.636663	0.751661	0.670001	0.045*	
C27	0.4657 (4)	0.8128 (3)	0.5282 (3)	0.0323 (7)	
H27	0.392058	0.803067	0.568133	0.039*	
C27A	0.4262 (3)	0.8595 (3)	0.4050 (3)	0.0269 (7)	0.81 (3)
N27A	0.4262 (3)	0.8595 (3)	0.4050 (3)	0.0269 (7)	0.19 (3)
Cd1	0.84479 (2)	0.18596 (2)	0.25036 (2)	0.02670 (9)	
Cl1	0.56777 (8)	0.18525 (7)	0.12937 (7)	0.03186 (16)	
Cl2	0.9928 (4)	0.2964 (3)	0.1472 (4)	0.0282 (3)	0.9008 (15)
I2	1.0135 (10)	0.3068 (9)	0.1370 (10)	0.0282 (3)	0.0992 (15)
Cl3	0.90087 (9)	−0.03537 (7)	0.23842 (8)	0.03669 (18)	
Cl4	0.89426 (8)	0.30982 (7)	0.47693 (7)	0.03601 (18)	

### Bis(2-methylimidazo[1,5-a]pyridinium) trichloridoiodidozincate(II) (III) Atomic displacement parameters (Å^2^)

**Table:**

	*U*^11^	*U*^22^	*U*^33^	*U*^12^	*U*^13^	*U*^23^
C11	0.0271 (14)	0.0234 (14)	0.0276 (15)	0.0046 (12)	0.0096 (11)	0.0023 (11)
N12	0.0249 (12)	0.0222 (12)	0.0287 (12)	0.0027 (10)	0.0110 (10)	0.0002 (10)
C12	0.0286 (15)	0.0320 (16)	0.0397 (17)	0.0046 (13)	0.0163 (13)	0.0038 (13)
C13	0.0292 (15)	0.0238 (14)	0.0282 (15)	0.0034 (12)	0.0083 (12)	0.0022 (11)
N13A	0.0297 (14)	0.0203 (13)	0.0311 (14)	0.0020 (10)	0.0122 (11)	−0.0002 (10)
C13A	0.0297 (14)	0.0203 (13)	0.0311 (14)	0.0020 (10)	0.0122 (11)	−0.0002 (10)
C14	0.0490 (19)	0.0291 (16)	0.0414 (18)	0.0038 (14)	0.0249 (15)	0.0095 (14)
C15	0.046 (2)	0.0317 (17)	0.054 (2)	−0.0027 (15)	0.0321 (17)	0.0006 (15)
C16	0.0281 (15)	0.0305 (16)	0.050 (2)	−0.0005 (13)	0.0182 (14)	−0.0065 (14)
C17	0.0281 (15)	0.0262 (15)	0.0329 (16)	0.0052 (12)	0.0102 (12)	−0.0040 (12)
C17A	0.0272 (14)	0.0210 (14)	0.0284 (15)	0.0040 (11)	0.0096 (11)	−0.0012 (11)
N17A	0.0272 (14)	0.0210 (14)	0.0284 (15)	0.0040 (11)	0.0096 (11)	−0.0012 (11)
C21	0.0288 (15)	0.0248 (15)	0.0349 (16)	0.0042 (12)	0.0133 (12)	−0.0035 (12)
N22	0.0377 (14)	0.0227 (12)	0.0269 (13)	0.0037 (11)	0.0085 (11)	−0.0015 (10)
C22	0.0459 (19)	0.0349 (18)	0.0314 (17)	0.0067 (15)	0.0033 (14)	0.0014 (14)
C23	0.0398 (17)	0.0233 (15)	0.0285 (15)	0.0008 (13)	0.0159 (13)	−0.0010 (12)
N23A	0.0334 (14)	0.0203 (12)	0.0285 (13)	0.0000 (10)	0.0156 (11)	−0.0031 (10)
C23A	0.0334 (14)	0.0203 (12)	0.0285 (13)	0.0000 (10)	0.0156 (11)	−0.0031 (10)
C24	0.0269 (15)	0.0257 (15)	0.0430 (18)	−0.0015 (12)	0.0172 (13)	−0.0080 (13)
C25	0.0332 (16)	0.0261 (16)	0.0409 (18)	0.0056 (13)	0.0058 (14)	−0.0027 (13)
C26	0.049 (2)	0.0265 (16)	0.0296 (16)	−0.0022 (14)	0.0083 (14)	0.0009 (13)
C27	0.0436 (18)	0.0245 (15)	0.0306 (16)	−0.0064 (13)	0.0192 (14)	−0.0029 (12)
C27A	0.0297 (15)	0.0222 (14)	0.0291 (15)	−0.0010 (11)	0.0147 (12)	−0.0038 (11)
N27A	0.0297 (15)	0.0222 (14)	0.0291 (15)	−0.0010 (11)	0.0147 (12)	−0.0038 (11)
Cd1	0.02659 (12)	0.02715 (13)	0.02568 (12)	0.00296 (8)	0.00879 (8)	0.00419 (8)
Cl1	0.0268 (3)	0.0371 (4)	0.0308 (4)	0.0035 (3)	0.0079 (3)	0.0094 (3)
Cl2	0.0328 (10)	0.0261 (7)	0.0321 (7)	0.0008 (6)	0.0181 (5)	0.0086 (4)
I2	0.0328 (10)	0.0261 (7)	0.0321 (7)	0.0008 (6)	0.0181 (5)	0.0086 (4)
Cl3	0.0409 (4)	0.0297 (4)	0.0459 (4)	0.0095 (3)	0.0204 (3)	0.0128 (3)
Cl4	0.0351 (4)	0.0413 (4)	0.0278 (4)	−0.0015 (3)	0.0094 (3)	0.0000 (3)

### Bis(2-methylimidazo[1,5-a]pyridinium) trichloridoiodidozincate(II) (III) Geometric parameters (Å, º)

**Table:**

C11—N12	1.362 (4)	C21—H21	0.9500
C11—N17A	1.363 (4)	N22—C23	1.338 (4)
C11—C17A	1.363 (4)	N22—C22	1.470 (4)
C11—H11	0.9500	C22—H22A	0.9800
N12—C13	1.338 (4)	C22—H22B	0.9800
N12—C12	1.462 (4)	C22—H22C	0.9800
C12—H12A	0.9800	C23—C23A	1.346 (4)
C12—H12B	0.9800	C23—N23A	1.346 (4)
C12—H12C	0.9800	C23—H23	0.9500
C13—C13A	1.347 (4)	N23A—C24	1.398 (4)
C13—N13A	1.347 (4)	N23A—C27A	1.399 (4)
C13—H13	0.9500	C23A—C24	1.398 (4)
N13A—C17A	1.402 (4)	C23A—N27A	1.399 (4)
N13A—C14	1.402 (4)	C24—C25	1.355 (5)
C13A—N17A	1.402 (4)	C24—H24	0.9500
C13A—C14	1.402 (4)	C25—C26	1.416 (5)
C14—C15	1.350 (5)	C25—H25	0.9500
C14—H14	0.9500	C26—C27	1.347 (5)
C15—C16	1.425 (5)	C26—H26	0.9500
C15—H15	0.9500	C27—N27A	1.420 (4)
C16—C17	1.348 (5)	C27—C27A	1.420 (4)
C16—H16	0.9500	C27—H27	0.9500
C17—N17A	1.413 (4)	Cd1—Cl3	2.4481 (8)
C17—C17A	1.413 (4)	Cd1—Cl2	2.4654 (16)
C17—H17	0.9500	Cd1—Cl4	2.4655 (7)
C21—N27A	1.360 (4)	Cd1—Cl1	2.4710 (7)
C21—C27A	1.360 (4)	Cd1—I2	2.747 (4)
C21—N22	1.364 (4)		
			
N12—C11—N17A	107.1 (3)	C23—N22—C22	124.4 (3)
N12—C11—C17A	107.1 (3)	C21—N22—C22	125.1 (3)
N12—C11—H11	126.4	N22—C22—H22A	109.5
C17A—C11—H11	126.4	N22—C22—H22B	109.5
C13—N12—C11	110.5 (2)	H22A—C22—H22B	109.5
C13—N12—C12	125.3 (3)	N22—C22—H22C	109.5
C11—N12—C12	124.2 (2)	H22A—C22—H22C	109.5
N12—C12—H12A	109.5	H22B—C22—H22C	109.5
N12—C12—H12B	109.5	N22—C23—C23A	107.4 (3)
H12A—C12—H12B	109.5	N22—C23—N23A	107.4 (3)
N12—C12—H12C	109.5	N22—C23—H23	126.3
H12A—C12—H12C	109.5	N23A—C23—H23	126.3
H12B—C12—H12C	109.5	C23—N23A—C24	130.1 (3)
N12—C13—C13A	107.3 (3)	C23—N23A—C27A	108.4 (3)
N12—C13—N13A	107.3 (3)	C24—N23A—C27A	121.5 (3)
N12—C13—H13	126.3	C23—C23A—C24	130.1 (3)
N13A—C13—H13	126.3	C23—C23A—N27A	108.4 (3)
C13—N13A—C17A	108.5 (2)	C24—C23A—N27A	121.5 (3)
C13—N13A—C14	131.1 (3)	C25—C24—N23A	118.1 (3)
C17A—N13A—C14	120.4 (3)	C25—C24—C23A	118.1 (3)
C13—C13A—N17A	108.5 (2)	C25—C24—H24	120.9
C13—C13A—C14	131.1 (3)	N23A—C24—H24	120.9
N17A—C13A—C14	120.4 (3)	C24—C25—C26	121.3 (3)
C15—C14—N13A	118.6 (3)	C24—C25—H25	119.3
C15—C14—C13A	118.6 (3)	C26—C25—H25	119.3
C15—C14—H14	120.7	C27—C26—C25	121.2 (3)
N13A—C14—H14	120.7	C27—C26—H26	119.4
C14—C15—C16	121.1 (3)	C25—C26—H26	119.4
C14—C15—H15	119.5	C26—C27—N27A	118.9 (3)
C16—C15—H15	119.5	C26—C27—C27A	118.9 (3)
C17—C16—C15	121.4 (3)	C26—C27—H27	120.5
C17—C16—H16	119.3	C27A—C27—H27	120.5
C15—C16—H16	119.3	C21—C27A—N23A	106.9 (3)
C16—C17—N17A	118.2 (3)	C21—C27A—C27	134.1 (3)
C16—C17—C17A	118.2 (3)	N23A—C27A—C27	119.0 (3)
C16—C17—H17	120.9	C21—N27A—C23A	106.9 (3)
C17A—C17—H17	120.9	C21—N27A—C27	134.1 (3)
C11—C17A—N13A	106.5 (2)	C23A—N27A—C27	119.0 (3)
C11—C17A—C17	133.2 (3)	Cl3—Cd1—Cl2	109.94 (9)
N13A—C17A—C17	120.3 (3)	Cl3—Cd1—Cl4	116.91 (3)
C11—N17A—C13A	106.5 (2)	Cl2—Cd1—Cl4	106.67 (9)
C11—N17A—C17	133.2 (3)	Cl3—Cd1—Cl1	105.93 (3)
C13A—N17A—C17	120.3 (3)	Cl2—Cd1—Cl1	112.21 (9)
N27A—C21—N22	106.9 (3)	Cl4—Cd1—Cl1	105.20 (3)
C27A—C21—N22	106.9 (3)	Cl3—Cd1—I2	109.2 (2)
C27A—C21—H21	126.6	Cl2—Cd1—I2	0.9 (3)
N22—C21—H21	126.6	Cl4—Cd1—I2	106.7 (2)
C23—N22—C21	110.5 (3)	Cl1—Cd1—I2	113.0 (2)
			
N17A—C11—N12—C13	−0.2 (3)	N27A—C21—N22—C23	0.5 (3)
C17A—C11—N12—C13	−0.2 (3)	C27A—C21—N22—C23	0.5 (3)
N17A—C11—N12—C12	178.3 (3)	N27A—C21—N22—C22	−178.3 (3)
C17A—C11—N12—C12	178.3 (3)	C27A—C21—N22—C22	−178.3 (3)
C11—N12—C13—C13A	−0.3 (3)	C21—N22—C23—C23A	−0.7 (3)
C12—N12—C13—C13A	−178.7 (2)	C22—N22—C23—C23A	178.1 (3)
C11—N12—C13—N13A	−0.3 (3)	C21—N22—C23—N23A	−0.7 (3)
C12—N12—C13—N13A	−178.7 (2)	C22—N22—C23—N23A	178.1 (3)
N12—C13—N13A—C17A	0.6 (3)	N22—C23—N23A—C24	179.8 (3)
N12—C13—N13A—C14	−177.6 (3)	N22—C23—N23A—C27A	0.5 (3)
N12—C13—C13A—N17A	0.6 (3)	N22—C23—C23A—C24	179.8 (3)
N12—C13—C13A—C14	−177.6 (3)	N22—C23—C23A—N27A	0.5 (3)
C13—N13A—C14—C15	178.5 (3)	C23—N23A—C24—C25	−179.5 (3)
C17A—N13A—C14—C15	0.4 (4)	C27A—N23A—C24—C25	−0.4 (4)
C13—C13A—C14—C15	178.5 (3)	C23—C23A—C24—C25	−179.5 (3)
N17A—C13A—C14—C15	0.4 (4)	N27A—C23A—C24—C25	−0.4 (4)
N13A—C14—C15—C16	0.7 (5)	N23A—C24—C25—C26	−1.0 (4)
C13A—C14—C15—C16	0.7 (5)	C23A—C24—C25—C26	−1.0 (4)
C14—C15—C16—C17	−0.9 (5)	C24—C25—C26—C27	1.4 (5)
C15—C16—C17—N17A	−0.2 (5)	C25—C26—C27—N27A	−0.4 (4)
C15—C16—C17—C17A	−0.2 (5)	C25—C26—C27—C27A	−0.4 (4)
N12—C11—C17A—N13A	0.5 (3)	N22—C21—C27A—N23A	−0.2 (3)
N12—C11—C17A—C17	179.7 (3)	N22—C21—C27A—C27	178.7 (3)
C13—N13A—C17A—C11	−0.7 (3)	C23—N23A—C27A—C21	−0.2 (3)
C14—N13A—C17A—C11	177.8 (3)	C24—N23A—C27A—C21	−179.6 (2)
C13—N13A—C17A—C17	−180.0 (2)	C23—N23A—C27A—C27	−179.3 (3)
C14—N13A—C17A—C17	−1.5 (4)	C24—N23A—C27A—C27	1.4 (4)
C16—C17—C17A—C11	−177.7 (3)	C26—C27—C27A—C21	−179.7 (3)
C16—C17—C17A—N13A	1.4 (4)	C26—C27—C27A—N23A	−1.0 (4)
N12—C11—N17A—C13A	0.5 (3)	N22—C21—N27A—C23A	−0.2 (3)
N12—C11—N17A—C17	179.7 (3)	N22—C21—N27A—C27	178.7 (3)
C13—C13A—N17A—C11	−0.7 (3)	C23—C23A—N27A—C21	−0.2 (3)
C14—C13A—N17A—C11	177.8 (3)	C24—C23A—N27A—C21	−179.6 (2)
C13—C13A—N17A—C17	−180.0 (2)	C23—C23A—N27A—C27	−179.3 (3)
C14—C13A—N17A—C17	−1.5 (4)	C24—C23A—N27A—C27	1.4 (4)
C16—C17—N17A—C11	−177.7 (3)	C26—C27—N27A—C21	−179.7 (3)
C16—C17—N17A—C13A	1.4 (4)	C26—C27—N27A—C23A	−1.0 (4)

### Bis(2-methylimidazo[1,5-a]pyridinium) trichloridoiodidozincate(II) (III) Hydrogen-bond geometry (Å, º)

**Table:**

*D*—H···*A*	*D*—H	H···*A*	*D*···*A*	*D*—H···*A*
C13—H13···Cl4	0.95	2.77	3.597 (3)	146
C12—H12*A*···Cl4^i^	0.98	2.71	3.520 (3)	141
C12—H12*C*···Cl2	0.98	2.76	3.652 (5)	152
C11—H11···Cl1^ii^	0.95	2.64	3.412 (3)	139
C17—H17···Cl1^ii^	0.95	2.81	3.560 (3)	137
C21—H21···Cl3^iii^	0.95	2.78	3.623 (3)	148
C23—H23···Cl1^iv^	0.95	2.83	3.447 (3)	123
C24—H24···Cl3^iv^	0.95	2.68	3.568 (3)	155
C27—H27···Cl4^v^	0.95	2.79	3.605 (3)	144

Symmetry codes: (i) −*x*+2, −*y*+1, −*z*+1; (ii) −*x*+1, −*y*+1, −*z*; (iii) *x*−1, *y*+1, *z*; (iv) *x*, *y*+1, *z*; (v) −*x*+1, −*y*+1, −*z*+1.
